# Supplementary material for: Filamentous fungal associates of the alder bark beetle, Alniphagus aspericollis, including an undescribed species of Neonectria
Source: PLoS One. 2023 May 8;18(5):e0284393. doi: 10.1371/journal.pone.0284393 (PMC10166519; doi:10.1371/journal.pone.0284393)
Supplement: S1 Table — Mycelia from each isolate comprising the listed strains, with the exception of several O. quercus isolates that were not successfully sub-cultured after initial investigation, were preserved in 10% glycerol, flash-frozen in liquid nitrogen, and placed at -80°C for long-term storage in the Genomics and Forest Health (Hamelin) Lab at the University of British Columbia, Canada. (DOCX) [file pone.0284393.s001.docx]

**Supporting Information**

**Table S1. Strains of Neonectria sp. nov., Ophiostoma quercus, Ophiostoma sp. nov., and Cadophora spadicis isolated from *Alniphagus aspericollis* and gallery phloem, including their collection site(s) and GenBank accession numbers for barcode sequences (internal transcribed spacer (ITS) rDNA gene region, partial beta-tubulin (BT) gene, partial translation elongation factor 1-alpha (EF1-α) gene, and partial RNA polymerase II second largest subunit (RPB2) gene).** Mycelia from each isolate comprising the listed strains, with the exception of several *O. quercus* isolates that were not successfully sub-cultured after initial investigation, were preserved in 10% glycerol, flash-frozen in liquid nitrogen, and placed at -80°C for long-term storage in the Genomics and Forest Health (Hamelin) Lab at the University of British Columbia, Canada.

| Species | Strain No. | Collection site(s) | GenBank accession No. (group ID) | | | |
| --- | --- | --- | --- | --- | --- | --- |
|  |  |  | **ITS** | **BT** | **EF1-α** | **RPB2** |
| Neonectria sp. nov. | CGL 401 | All sites | OP787855 (NEO01) | - | OP739237 (NEOE1) | OP739236 (NEOR1) |
|  | CGL 402 | GP | OP787854  (NEO01) | - | OP739235 (NEOE2) | OP739234 (NEOR2) |
| Ophiostoma quercus | CGL 101 | CM, PS, BM | OP787853 (OPQ01) | OP739233 (OPQB1) | OP739232 (OPQE1) | - |
|  | CGL 102 | CM, TH, GP | OP787852 (OPQ01) | OP739231 (OPQB1) | OP739230 (OPQE2) | - |
|  | CGL 103 | PS | OP787851 (OPQ02) | OP739229 (OPQB2) | OP739228 (OPQE3) | - |
|  | CGL 104 | PS | OP787850 (OPQ02) | OP739227 (OPQB2) | OP739226 (OPQE4) | - |
|  | CGL 105 | PS | OP787849 (OPQ02) | OP739225 (OPQB2) | OP739224 (OPQE5) | - |
|  | CGL 106 | PS | OP787848 (OPQ01) | OP739223 (OPQB1) | OP739222 (OPQE6) | - |
|  | CGL 107 | TH | OP787847 (OPQ01) | OP739221 (OPQB1) | OP739220 (OPQE7) | - |
|  | CGL 108 | TH | OP787846 (OPQ01) | OP739219 (OPQB1) | OP739218 (OPQE8) | - |
|  | CGL 109 | BL | OP787845 (OPQ01) | OP739217 (OPQB1) | OP739216 (OPQE9) | - |
|  | CGL 110 | BM | OP787844 (OPQ01) | OP739215 (OPQB3) | OP739214 (OPQE1) | - |
|  | CGL 111 | TH | OP787843 (OPQ01) | OP739213 (OPQB4) | OP739212 (OPQE2) | - |
|  | CGL 112 | TH | OP787842  (OPQ03) | OP739211 (OPQB1) | OP739210 (OPQE2) | - |
|  | CGL 113 | BL | OP787841  (OPQ03) | OP739209 (OPQB1) | OP739208 (OPQE1) | - |
| Ophiostoma sp. nov. | CGL 201 | BM | OP787840  (OPS1) | OP739207 (OPSB1) | OP739206 (OPSE1) | - |
| Cadophora spadicis | CGL 301 | AG, BM, CM | OP787862 (CAD01) | OP739243 (CADB1) | OP739242 (CADE1) | - |
|  | CGL 302 | TH | OP787861 (CAD02) | OP739241 (CADB1) | OP739240 (CADE1) | - |
|  | CGL 303 | AG | OP787860  (CAD01) | OP739239 (CADB2) | OP739238 (CADE2) | - |

Sampling site legend: CM = Cypress Mountain; PS = Pacific Spirit Regional Park; TH = Tynehead Regional Park; AG = Aldergrove Regional Park; GP = Gates Park; BL = Burnaby Lake Regional Park; BM = Burnaby Mountain Conservation Area.
