# Supplementary material for: Filamentous fungal associates of the alder bark beetle, Alniphagus aspericollis, including an undescribed species of Neonectria
Source: PLoS One. 2023 May 8;18(5):e0284393. doi: 10.1371/journal.pone.0284393 (PMC10166519; doi:10.1371/journal.pone.0284393)

PLOS ONE

A novel type of bark beetle–fungus association between the hardwood-killing alder bark beetle, Alniphagus aspericollis, and a Neonectria canker pathogen

--Manuscript Draft--

**Manuscript Number:**	PONE-D-22-28596
**Article Type:**	Research Article
**Full Title:**	A novel type of bark beetle–fungus association between the hardwood-killing alder bark beetle, Alniphagus aspericollis, and a Neonectria canker pathogen
**Short Title:**	The alder bark beetle is associated with a Neonectria canker pathogen
**Corresponding Author:**	Allan L. Carroll, Ph.D.<br>The University of British Columbia Faculty of Forestry<br>Vancouver, BC CANADA
**Keywords:**	Scolytinae; insect-plant interactions; bark beetle-fungus associations; insect-fungal mutualisms; symbioses; bark beetle-angiosperm interactions; canker pathogen
**Abstract:**	Bark beetles (Coleoptera: Curculionidae; Scolytinae) are tree-infesting insects that consume subcortical tissues and fungi. Species capable of killing their host trees are most commonly associated with conifers, as very few bark beetle species infest and kill hardwood hosts directly. The alder bark beetle, Alniphagus aspericollis, is a hardwood-killing bark beetle that colonizes and kills red alder, Alnus rubra. Conifer-killing bark beetles have well-known associations with symbiotic ophiostomatoid fungi that facilitate their life histories, but it is unknown whether A. aspericollis has any fungal associates. This study was conducted to identify any consistent filamentous fungal associates of A. aspericollis and characterize the consistency of observed beetle–fungus relationships. Beetles and gallery phloem samples were collected from seven sites throughout the Greater Vancouver region in British Columbia, Canada. Filamentous fungi were isolated from these samples and identified by DNA barcoding using the internal transcribed spacer (ITS) region and other barcode regions for resolution to the species-level for the most dominant isolates. The most common fungal associate was a previously undescribed Neonectria major-like canker pathogen, Neonectria sp. nov., which was isolated from ~67% of adult beetles, ~59% of phloem samples, and ~94% of the beetle-infested trees. Ophiostoma quercus was isolated from ~28% of adult beetles, ~9% of phloem samples, and ~56% of infested trees and deemed a casual associate of A. aspericollis, while a putatively novel species of Ophiostoma was more infrequently isolated from A. aspericollis and its galleries. Cadophora spadicis, a new decay pathogen record for red alder, was rarely isolated and is probably coincidentally carried by A. aspericollis. Overall, A. aspericollis was only loosely associated with ophiostomatoid fungi, suggesting that these fungi have little ecological significance in the beetle–tree interaction, while Neonectria sp. nov. may be a symbiote of A. aspericollis that is vectored by the beetle.
**Order of Authors:**	Gervais Y.S. Lee, B.SC.
	Debra L. Wertman, M.Sc.
	Allan L. Carroll, Ph.D.
	Richard C. Hamelin, Ph.D.
**Additional Information:**	
**Question**	**Response**
**Financial Disclosure**<br><br>Enter a financial disclosure statement that describes the sources of funding for the work included in this submission. Review the submission guidelines for detailed requirements. View published research	ALC: RGPIN-2015-04376, Natural Sciences and Engineering Research Council of Canada, Discovery Grant, https://www.nserc-crsng.gc.ca/<br><br>RCH: RGPIN-2015-05279, Natural Sciences and Engineering Research Council of Canada, Discovery Grant, https://www.nserc-crsng.gc.ca/<br><br>The funding agency did not play any role in the study design, data collection and

Powered by Editorial Manager® and ProduXion Manager® from Aries Systems Corporation

articles from *PLOS ONE* for specific examples.<br><br>This statement is required for submission and **will appear in the published article** if the submission is accepted. Please make sure it is accurate.<br><br>**Unfunded studies**<br>Enter: *The author(s) received no specific funding for this work.*<br><br>**Funded studies**<br>Enter a statement with the following details:<br>• Initials of the authors who received each award<br>• Grant numbers awarded to each author<br>• The full name of each funder<br>• URL of each funder website<br>• Did the sponsors or funders play any role in the study design, data collection and analysis, decision to publish, or preparation of the manuscript?<br>• **NO** - Include this sentence at the end of your statement: *The funders had no role in study design, data collection and analysis, decision to publish, or preparation of the manuscript.*<br>• **YES** - Specify the role(s) played.<br><br>* typeset	analysis, decision to publish, or preparation of the manuscript.
**Competing Interests**<br><br>Use the instructions below to enter a competing interest statement for this submission. On behalf of all authors, disclose any competing interests that could be perceived to bias this work—acknowledging all financial support and any other relevant financial or non-financial competing interests.<br><br>This statement is required for submission and **will appear in the published article** if the submission is accepted. Please make sure it is accurate and that any funding sources listed in your Funding Information later in the submission form are also declared in your Financial Disclosure statement.	The authors have declared that no competing interests exist.

View published research articles from *PLOS ONE* for specific examples.<br><br>**NO authors have competing interests**<br><br>Enter: *The authors have declared that no competing interests exist.*<br><br>**Authors with competing interests**<br><br>Enter competing interest details beginning with this statement:<br><br>*I have read the journal's policy and the authors of this manuscript have the following competing interests: [insert competing interests here]*<br><br>* typeset	
**Ethics Statement**<br><br>Enter an ethics statement for this submission. This statement is required if the study involved:<br><br>• Human participants<br>• Human specimens or tissue<br>• Vertebrate animals or cephalopods<br>• Vertebrate embryos or tissues<br>• Field research<br><br>Write "N/A" if the submission does not require an ethics statement.<br><br>General guidance is provided below. Consult the submission guidelines for detailed instructions. **Make sure that all information entered here is included in the Methods section of the manuscript.**	N/A

**Format for specific study types**<br><br>**Human Subject Research (involving human participants and/or tissue)**<br>• Give the name of the institutional review board or ethics committee that approved the study<br>• Include the approval number and/or a statement indicating approval of this research<br>• Indicate the form of consent obtained (written/oral) or the reason that consent was not obtained (e.g. the data were analyzed anonymously)<br><br>**Animal Research (involving vertebrate animals, embryos or tissues)**<br>• Provide the name of the Institutional Animal Care and Use Committee (IACUC) or other relevant ethics board that reviewed the study protocol, and indicate whether they approved this research or granted a formal waiver of ethical approval<br>• Include an approval number if one was obtained<br>• If the study involved *non-human primates*, add *additional details* about animal welfare and steps taken to ameliorate suffering<br>• If anesthesia, euthanasia, or any kind of animal sacrifice is part of the study, include briefly which substances and/or methods were applied<br><br>**Field Research**<br><br>Include the following details if this study involves the collection of plant, animal, or other materials from a natural setting:<br>• Field permit number<br>• Name of the institution or relevant body that granted permission	
**Data Availability**<br><br>Authors are required to make all data underlying the findings described fully available, without restriction, and from the time of publication. PLOS allows rare exceptions to address legal and ethical concerns. See the PLOS Data Policy and FAQ for detailed information.	Yes - all data are fully available without restriction

A Data Availability Statement describing where the data can be found is required at submission. Your answers to this question constitute the Data Availability Statement and **will be published in the article**, if accepted.<br><br>**Important:** Stating 'data available on request from the author' is not sufficient. If your data are only available upon request, select 'No' for the first question and explain your exceptional situation in the text box.<br><br>Do the authors confirm that all data underlying the findings described in their manuscript are fully available without restriction?	
**Describe where the data may be found in full sentences. If you are copying our sample text, replace any instances of XXX with the appropriate details.**<br><br>• If the data are **held or will be held in a public repository**, include URLs, accession numbers or DOIs. If this information will only be available after acceptance, indicate this by ticking the box below. For example: *All XXX files are available from the XXX database (accession number(s) XXX, XXX.)*.<br>• If the data are all contained **within the manuscript and/or Supporting Information files**, enter the following: *All relevant data are within the manuscript and its Supporting Information files.*<br>• If neither of these applies but you are able to provide **details of access elsewhere**, with or without limitations, please do so. For example:<br><br>*Data cannot be shared publicly because of [XXX]. Data are available from the XXX Institutional Data Access / Ethics Committee (contact via XXX) for researchers who meet the criteria for access to confidential data.*<br><br>*The data underlying the results presented in the study are available from (include the name of the third party*	All relevant data are within the manuscript and its Supporting Information files.

and contact information or URL).<br>• This text is appropriate if the data are owned by a third party and authors do not have permission to share the data.<br><br>* typeset	
Additional data availability information:	

Manuscropt
Click here to access/download;Manuscript;Lee et al_PLOS ONE submitted.docx

A novel type of bark beetle–fungus association between the hardwood-killing alder bark beetle, *Alniphagus aspericollis*, and a *Neonectria* canker pathogen

Short title: The alder bark beetle is associated with a *Neonectria* canker pathogen

Gervais Y.S. Lee[1,#a], Debra L. Wertman[1], Allan L. Carroll[1*], and Richard C. Hamelin[1]

[1] Department of Forest and Conservation Sciences, The University of British Columbia, Vancouver, British Columbia, Canada

[#a] Current Address: Singapore Botanic Gardens, National Parks Board, Singapore

[*] Corresponding author

E-mail: allan.carroll@ubc.ca (ALC)

Author Contributions

Conceptualization: ALC, RCH, GYSL; experiments performed by: GYSL; Data analysis: GYSL, DLW; Reagents, materials and analysis tools contributed by: RCH; Writing: GYSL, DLW, RCH and ALC.

Abstract

Bark beetles (Coleoptera: Curculionidae; Scolytinae) are tree-infesting insects that consume subcortical tissues and fungi. Species capable of killing their host trees are most commonly associated with conifers, as very few bark beetle species infest and kill hardwood hosts directly. The alder bark beetle, *Alniphagus aspericollis*, is a hardwood-killing bark beetle that colonizes and kills red alder, *Alnus rubra*. Conifer-killing bark beetles have well-known associations with symbiotic ophiostomatoid fungi that facilitate their life histories, but it is unknown whether *A. aspericollis* has any fungal associates. This study was conducted to identify any consistent filamentous fungal associates of *A. aspericollis* and characterize the consistency of observed beetle–fungus relationships. Beetles and gallery phloem samples were collected from seven sites throughout the Greater Vancouver region in British Columbia, Canada. Filamentous fungi were isolated from these samples and identified by DNA barcoding using the internal transcribed spacer (ITS) region and other barcode regions for resolution to the species-level for the most dominant isolates. The most common fungal associate was a previously undescribed *Neonectria major*-like canker pathogen, *Neonectria* sp. nov., which was isolated from ~67% of adult beetles, ~59% of phloem samples, and ~94% of the beetle-infested trees. *Ophiostoma quercus* was isolated from ~28% of adult beetles, ~9% of phloem samples, and ~56% of infested trees and deemed a casual associate of *A. aspericollis*, while a putatively novel species of *Ophiostoma* was more infrequently isolated from *A. aspericollis* and its galleries. *Cadophora spadicis*, a new decay pathogen record for red alder, was rarely isolated and is probably coincidentally carried by *A. aspericollis*. Overall, *A. aspericollis* was only loosely associated with ophiostomatoid fungi, suggesting that these fungi have little ecological

significance in the beetle–tree interaction, while *Neonectria* sp. nov. may be a symbiote of *A. aspericollis* that is vectored by the beetle.

Introduction

Bark beetles of the subfamily Scolytinae (Coleoptera: Curculionidae) are subcortical herbivore-fungivores that occur in forested ecosystems worldwide. They range from monophagous to oligophagous, breeding in the phloem-cambium tissues of their tree hosts where their larvae feed and pupate [1,2]. Most bark beetles are secondary invaders and only target stressed or moribund trees that have been weakened by other biotic or abiotic stressors, such as defoliators and drought [3-5]. Some bark beetle species, however, occasionally build epidemic populations that overwhelm healthy, live trees via pheromone-mediated mass attacks [4,6]. These irruptive bark beetles have historically caused widespread tree-mortality events at the landscape scale, resulting in significant economic and ecological consequences [7-11].

Bark beetles evolved alongside their symbiotic fungal partners [12]. These fungi may either be carried phoretically on the exoskeleton of the beetles [13-15] or contained within specialised structures called mycangia that occur within the mouthparts, thorax, or elytra of the beetles [16-18]. Bark beetle–fungus relationships have typically been considered as mutually beneficial to both the beetle and fungus partners [19]. Beetles facilitate the transmission of fungi from one host tree to another, thereby enabling them to colonise fresh resources ahead of other late successional saprophytes [20]. In return, beetles benefit from improved nutrition to developing larvae within fungal-colonized phloem [21-23], and/or phytopathogenic activity by fungi that exhausts host tree defenses [24] and kills them by blocking water conduction in the xylem [5,25,26]. Reviews by Paine et al. [5] and Six and Wingfield [20] have highlighted that

not all bark beetle–fungus relationships are necessarily mutualistic. Rather, these symbioses comprise a spectrum ranging from true mutualisms, to commensalism, or even antagonistic associations that can be obligatory, facultative, or purely coincidental [20]. The nature of bark beetle–fungus relationships apparently vary relative to host tree vigor, the specific fungal associate, and the life history of the beetle [5,27]. The most well-studied bark beetle-symbiotic fungi are found in conifer systems and belong to the ascomycete order Ophiostomatales, comprising the genera *Ophiostoma*, *Grosmannia*, *Leptographium*, and *Raffaelea* [20,28-30]. The genera *Ceratocystis* and *Bretziella* (Order: Microascales) also contains “ophiostomatoid” fungi associated with bark beetles.

The majority of bark beetle species capable of infesting and killing their host trees across landscapes are associated with conifers [31]. Most bark beetles that feed on hardwoods are not stand-level tree killers and instead colonise moribund hosts or dying tree limbs. Even in the well-known cases of Dutch elm disease, *Ophiostoma ulmi* and *O. novo-ulmi*, and oak wilt, *Bretziella fagacearum*, that are associated with the bark beetles *Scolytus* spp. and *Pseudopityophthorus* spp. respectively, healthy host trees are first infected with the virulent pathogens during adult beetle maturation feeding (e.g. in branch crotches), and subcortical invasion by the beetles does not occur until weeks after initial inoculation, when the host tree is already dying from the disease [32-34]. Ohmart [31] hypothesized that the evolution of hardwood-killing bark beetles might have been hampered, either because induced defenses of hardwoods were too complex, or because the nutritional benefits gained by hardwood-invading bark beetles were outweighed by the high physiological costs of colonization. Given that the fungal symbionts of bark beetles may play a role in overcoming host tree defenses, examining the fungal communities of the few apparent hardwood-killing beetles could provide insight into how these beetles kill trees.

The alder bark beetle, *Alniphagus aspericollis* (LeConte) attacks red alder, *Alnus rubra* (Bong.), an early successional nitrogen-fixing hardwood found throughout the coastal Pacific Northwest [35-38]. Though *A. aspericollis* is frequently found in alder slash, it is able to kill live red alder that is either over-mature or has been previously stressed by other factors [35,36]. Symbiotic fungal partners of *A. aspericollis*, if any, are yet to be discovered. Borden [36] observed that infestation by *A. aspericollis* could precede wood rot but did not suggest that fungal inoculation was directly or indirectly facilitated by *A. aspericollis*. Previous studies of the fungal communities of red alder in the Pacific Northwest have recorded various species of decay and non-ophiostomatoid pathogenic fungi but no associations with *A. aspericollis* were reported [39,40].

This study explores the filamentous fungal community associated with *A. aspericollis*. Our objectives were to determine if *A. aspericollis* is associated with fungi and characterize the consistency of any beetle-fungus relationships. Given the ability of *A. rubra* to fix nitrogen from the atmosphere via symbiotic root nodule actinomycetes [37,38], we also tested the prediction that *A. aspericollis* would not require, and therefore not be consistently associated with, nitrogen-provisioning ophiostomatoid fungi in the same manner as conifer-feeding bark beetles that depend on these fungi to complete their development [21-23].

Methods

Beetle and gallery phloem collection

Adult *A. aspericollis* (Fig 1) were collected from seven sites across the Greater Vancouver region in British Columbia, Canada between 14 July and 26 August 2015 (Fig 2). One to three infested red alder hosts were located at each site, and three adult beetles were

obtained from under the bark of each tree (Figs 1 and 2). Individual beetles were extracted from separate entrance holes and non-overlapping galleries. Larvae, pupae and teneral adults were also collected when present. All specimens were individually transferred into separate vials with ethanol-sterilized tweezers. A section of phloem (approximately 5 x 5cm) was also extracted from each of the sampled beetle galleries using a hammer and chisel. All beetle and gallery phloem samples were transported back to the laboratory and refrigerated until fungal isolations were conducted.

Fig 1. The alder bark beetle, *Alniphagus aspericollis*, and its injuries on host red alder, *Alnus rubra*. (A) Lateral view of an adult; (B) maternal gallery (eggs, including one dislodged from its niche at the top of the gallery, are indicated by arrows); (C) attack initiation; (D) newly-constructed gallery entrance holes in the bark of an infested tree; (E) maternal gallery and outward-radiating larval galleries in early stages of construction, with associated phloem staining; and (F) typical red alder stand where *A. aspericollis*-infested trees are located.

Fig 2. Sampling locations within the Greater Vancouver region, British Columbia, Canada; (1) Cypress Mountain, (2) Pacific Spirit Regional Park, (3) Tynehead Regional Park, (4) Aldergrove Regional Park, (5) Gates Park, (6) Burnaby Lake Regional Park, and (7) Burnaby Mountain Conservation Area. Map obtained from DataBC, Province of British Columbia, Canada (2003). Three adult *Alniphagus aspericollis* were collected from each tree, with the number of trees sampled (N) per site indicated on the map. Specific locations and sampling dates for each site are as follows: (1) 49°21'34" N, 123°12'38" W, 14 July 2015; (2) 49°16'16" N, 123°14'20" W, 23 July 2015; (3) 49°10'49" N, 122°45'54" W, 22 July 2015; (4)

49°0'56" N, 122°27'58" W, 22 July 2015; (5) 49°15'24" N, 122°47'30" W, 24 August 2015; (6) 49°14'46" N, 122°55'50" W, 26 August 2015; (7) 49°16'50" N, 122°55'12" W, 26 August 2015.

Fungal isolations

Fungal isolations from the beetle and phloem samples were performed within one week of collection. Each beetle was thoroughly washed with 100µl of sterile water and 50µl of the resulting liquid inoculum was thinly spread onto malt extract agar (MEA) in a petri dish. From each phloem sample, five thinly-sliced chips (approximately 1 x 1cm) were sectioned out with sterilized instruments, particularly around the entrance hole and gallery regions. Phloem chips were rapidly surface sterilized using 70% ethanol, rinsed thoroughly with sterile water, and plated onto water agar in a petri dish for incubation at room temperature.

Individual filamentous fungal colonies emerged in the initial isolation dishes within three days of incubation. Since the colonies in each dish were generally too numerous to be individually isolated, they were first sorted into different morphotypes on the basis of visible characteristics (e.g. colony diameter, texture, hyphal/spore colour, margin characteristics). Representative samples and variants of each morphotype were then sub-cultured onto MEA to ensure that maximum fungal diversity was captured per initial isolation dish. Sub-cultures were incubated at room temperature, and repeatedly sub-cultured until pure isolates were obtained. Numerous yeast and bacterial colonies were also observed in most of the initial isolation dishes but were not sub-cultured as these taxa were beyond the scope of this study.

DNA extraction, amplification, and sequencing

Mycelia from each pure isolate were frozen via liquid nitrogen and finely-ground. DNA samples were extracted with the Qiagen Dneasy® Plant Mini Kit (Qiagen Inc., Valencia, California, USA) according to the manufacturer's instructions. Internal transcribed spacer (ITS) rDNA gene region, which is a universal DNA barcode for all fungi species [41], was amplified from all samples using the primers ITS1F and ITS4 [42,43]. PCR was performed with the ABI 2720 Thermal Cycler (Applied Biosystems, Foster City, California, USA) or the T100™ Thermal Cycler (Bio-Rad, Hercules, California, USA) using a 27µl reaction system and the following thermal cycler program: 94°C for 3 min, followed by 35 cycles of 94°C for 30 s, 55°C for 30 s, 72°C for 1 min, and then held for 10 min at 72°C. PCR amplicons were purified using Unifilter™ GF/C 384-well 100µl microplates (Whatman Inc., Ann Arbor, Michigan, USA) and Sanger sequenced with the ABI 3730xl DNA Sequencer (Applied Biosystems, Foster City, California, USA) at the Centre de Recherche du CHUL (CHUQ), Université Laval (Quebec, Canada). ITS1F was used as the forward sequencing primer to produce single-strand sequences for all amplicons.

Additional gene regions were amplified and sequenced to confirm species identities for the fungal morphotypes most frequently isolated from beetles and phloem. For isolates from the genus *Neonectria*, two gene regions were targeted: i) the partial RNA polymerase II second largest subunit (RPB2) gene, amplified using nRPB2-147 and fRPB2-7cR primers [44,45] and forward sequenced using nPRB2-147, and ii) the partial translation elongation factor 1-alpha (EF1-α) gene, amplified using EF1-728F and EF1-986RN primers [46,47] and forward sequenced using EF1-728F. For isolates from the genus *Ophiostoma*, target regions included i) the partial beta-tubulin (BT) gene, amplified using Bt2A and Bt2B primers [48] and forward

sequenced using Bt2A, and ii) the partial translation elongation factor 1-alpha (EF1-α) gene, amplified using EF1-728F and EF1-986R primers [46] and forward sequenced using EF1-728F. Targeted gene regions for isolates from the genus *Cadophora* were i) the partial translation elongation factor 1-alpha (EF1-α) gene, amplified using EF1-688F and EF1-1251R primers [49] and forward sequenced using EF1-688F, and ii) the partial beta-tubulin (BT) gene, amplified using BTCadF and BTCadR [50] and forward sequenced using BTCadF.

Sequence analysis and fungal identification

Raw sequences were edited and verified using BioEdit Sequence Alignment Editor software [51] version 7.1 (2011). Resulting ITS sequences were compared to existing GenBank sequence data using a BLAST similarity search (BLASTn) to identify each isolate to the genus- or species-level. To verify the species identities of the most frequently isolated morphotypes, single-locus phylogenetic trees were constructed via maximum likelihood analysis, using PhyML 3.0 [52] with the HKY85 substitution model. Phylogenies were constructed for each *Neonectria*, *Ophiostoma*, and *Cadophora* gene region. The species identity of each frequently-isolated morphotype was determined based on its phylogenetic placement relative to other closely-related species. Comparative phylogenetic data was derived from the most current relevant studies that also provided appropriate outgroups for tree rooting. For *Neonectria* isolates, *N. castaneicola* was chosen as the outgroup for the ITS dataset [45], while the more related *N. fuckeliana* was selected as the outgroup for the RPB2 dataset [47].

Results

Fungi associated with *A. aspericollis* were identified to the genus or species-level for 89% of the fungal cultures obtained, based on DNA sequence homology of ≥ 99%. Comparative homology assessment of ITS barcode sequences identified fungi belonging to the genera *Neonectria*, *Ophiostoma*, *Graphilbum*, *Cadophora*, *Penicillium*, *Beauveria*, *Cladosporium*, *Acremonium*, *Cosmospora*, *Chondrostereum*, *Valsalnicola*, *Pezicula*, and *Umbelopsis* (Table 1). Lower DNA sequence homology matches (94-97%) were obtained for ITS sequences from fungal cultures of unknown Ascomycota (Table 2).

Table 1. GenBank accession numbers for the ITS barcode sequences of filamentous fungi associated with *Alniphagus aspericollis*, corresponding closest BLAST (BLASTn) matches in GenBank and percent sequence homology.

**Fungus**	**Accession No. (sequence ID)**	**Closest BLAST matches**	**Accession no. of closest matches**	**% similarity**
**Canker pathogens**				
Neonectria sp. nov.	(NEO01)	*N. major*, type material	NR_121496	99
		“*N. galligena*”/*N. ditissima* [a]	JQ434582	99
		N. major	JF268767	99
		N. ditissima	HQ166494	99
**Ophiostomatoid fungi**				

Ophiostoma quercus	(OPQ01)	*O. quercus*	JX444663	100
		O. quercus	JQ319590	100
	(OPQ02)	*O. piceae*	KF531618	100
		O. quercus	KM100571	100
		O. quercus	FJ805463	100
	(OPQ03)	*O. quercus*	JX444663	99
		O. quercus	JQ319590	99
Ophiostoma sp. nov.	(OPS01)	*O. catonianum*	EU443765	98
		O. piceae	KF531618	98
		O. quercus	KM100571	98
Graphilbum fragrans	(GRA01)	"*Pesotum fragrans*"/ *G. fragrans* [b]	JX444673	100
		P. fragrans	DQ396790	100
		"*Ophiostoma microcarpum*"/ *G. microcarpum* [c]	GU134170	100
**Other fungi**				
Cadophora spadicis	(CAD01)	*C.* sp.	HQ713754	100
		"*C. melinii*"/*C. spadicis* [d]	DQ404351	100
		C. spadicis	KM497029	100
		C. malorum	FJ903289	100
	(CAD02)	*C.* sp.	HQ713754	99
		"*C. melinii*"/*C. spadicis* [d]	DQ404351	99

		C. spadicis	KM497029	99
		C. malorum	FJ903289	99
Beauveria spp.	(BEA01)	*B. bassiana*	HQ880760	100
		B. sp.	GQ354221	100
	(BEA02)	*B. bassiana*	KP670430	99
		B. sp.	KF367502	99
		B. pseudobassiana	AB831659	99
Chondrostereum purpureum	(CHO01)	*C. purpureum*	KP195081	99
		C. purpureum	KT362916	99
Valsalnicola oxystoma	(VAL01)	*V. oxystoma*	JX519559	100
Penicillium spp.	(PEN01)	*P. hoeksii*	KM189523	100
		P. zhuangii	KF769435	99
		P. quercetorum	KM189556	99
	(PEN02)	*P. pancosmium*	KP329841	100
		P. sp.	KF367512	100
		P. waksmanii	HQ607920	100
	(PEN03)	*P. waksmanii*	AY373940	100
		P. ubiquetum	JN617679	100
	(PEN04)	*P. nothofagi*, type material	NR_121518	100
		P. godlewskii, type material	NR_103658	100
		P. cosmopolitanum	JN617682	100

		P. canescens	AF034463	100
	(PEN05)	*P. westlingii*	JN617668	100
		P. decaturense	KT323158	100
	(PEN06)	*P. vancouverense*, type material	NR_121512	100
		P. wellingtonense, type material	NR_121519	99
		P. pasqualense, type material	NR_121513	99
	(PEN07)	*P. brevicompactum*	KP903609	100
		P. bialowiezense	KC427176	100
		P. biourgeianum	JX139727	100
	(PEN08)	*P.* sp.	KT121512	100
		P. brevicompactum	KP329602	100
	(PEN09)	*P. glabrum*	KR909187	100
		P. spinulosum	KF646101	100
		P. adametzioides	DQ681325	100
	(PEN10)	*P. kojigenum*, type material	NR_121253	100
		P. sp.	AM901674	100
Cladosporium sp.	(CLA01)	*C. cladosporioides*	KT824762	100
		C. sp.	KT315420	100
		C. subuliforme	KT600456	100

		C. montecillanum	KT600408	100
Acremonium sp.	(ACR01)	*A. furcatum*	JF311939	99
		Hypocreales sp.	HQ649873	99
		A. furcatum, type strain	AY378154	99
Cosmospora sp.	(COS01)	"*Acremonium* cf. *curvulum*" [e]	KM231818	100
		C. viridescens	JQ676175	100
		C. viridescens [f]	KJ676147	99
Pezicula sp.	(PEZ01)	*P. sporulosa*	KR859265	99
		P. livida	AF141180	99
		P. californiae	JX144758	99
Umbelopsis sp.	(UMB01)	*U. ramanniana*	KM017730	100
		U. isabellina	HQ630363	99
Unknown Ascomycota spp.	(ASC01)	Roussollaceae sp.	KR014367	97
		Ascomycota sp.	KT004570	94
	(ASC02)	Ascomycete sp.	EU816396	96
		Ascomycota sp.	KF998994	95

[a] "*Neonectria galligena*" is now synonymous with *N. ditissima*, with the latter recognised as the official name [47].

[b] Renamed as *Graphilbum fragrans* [29].

[c] Renamed as *Graphilbum microcarpum* [29].

[d] Erroneously named as *C. melinii* by [53], corrected by [50] as *C. spadicis*.

[e] Placed into the genus *Cosmospora* by [54].

[f] From the phylogenetic study of *Cosmospora* by [55].

Phylogenetic analyses and species identification

Isolates from the genus *Neonectria* (strains CGL 401 and CGL 402) were identified as an undescribed *N. major*-like species, hereafter *Neonectria* sp. nov., based upon morphological culture characteristics, existing host-specificity data, and by comparison of ITS, RPB2, and EF1-α sequences with those of closely-related *Neonectria* species (Fig 3) [45,47,56]. Overall, our phylogenetic analyses placed these *Neonectria* isolates most closely to *N. major* and *N. ditissima* but within a well-supported distinct clade (88-98% bootstrap support). DNA amplification of *N.* sp. nov. isolates resulted in fragments of ~500bp for the ITS barcode region, ~960bp for the partial RPB2 gene region, and ~260bp for the partial EF1-α gene region. The identity of *N*. sp. nov. could not be fully resolved by phylogenetic analysis of the ITS barcode region, which grouped the isolates with species such as *N. major*, *N. ditissima,* and *N. neomacrospora* (Fig 3A). Phylogenetic analysis of the partial RPB2 gene region placed the *N*. sp. nov. isolates within a single well-supported clade that was most closely related to *N. major* and *N. ditissima*, thereby distinguishing the species from *N. neomacrospora* and *N. ditissimopsis* (Fig 3B). Analysis of the partial EF1-α gene region also placed the isolates within a single well-supported clade that was related to, but distinct from, *N. ditissimopsis* and slightly more distantly related to *N. major* and *N. ditissima* (Fig 3C). *Neonectria major,* but not *N. ditissima*, has been isolated from *Alnus* hosts and the cultural characteristics of our *N*. sp. nov. isolates (e.g., regularly-tufted aerial mycelia, saffron pigmentation on underside) (Fig 4A) are in agreement with existing descriptions of *N. major* [47], leading us to conclude that the *N*. sp. nov. isolates observed during this study are *N. major*-like.

Fig 3. Single-locus maximum likelihood phylogenetic trees showing the phylogenetic placement of *Neonectria* sp. nov. isolates associated with *Alniphagus aspericollis* (marked by black arrows); (A) ITS region, (B) partial RPB2 region, and (C) partial EF1-α region. Only maximum likelihood values that exceed 0.65 are shown among the branches and are indicated by bold lines. GenBank accession numbers of previously published *Neonectria* spp. sequences are included.

Fig 4. Front and reverse sides of malt extract agar cultures of various filamentous fungi associated with *Alniphagus aspericollis*. (A) *Neonectria* sp. nov (*N. major*-like), (B,C) *Ophiostoma quercus*, (D) *Ophiostoma* sp. nov., (E) *Graphilbum fragrans*, and (F) *Cadophora spadicis*. Cultures were photographed following variable growth periods.

Isolates from the genus *Ophiostoma* were identified to the species-level based upon comparison of ITS, BT and EF1-α sequences with those of closely-related species [57-63] in the "*O. ulmi* complex" [64]. DNA amplification of *Ophiostoma* isolates resulted in fragments of ~600bp for the ITS barcode region, ~400bp for the partial BT gene region, and ~460-540bp for the partial EF1-α gene region. Phylogenetic analyses conducted for all three gene regions split the *Ophiostoma* isolates into two distinct groups (Fig 5).

Fig 5. Single-locus maximum likelihood phylogenetic trees indicating the phylogenetic placement of *Ophiostoma* strains associated with *Alniphagus aspericollis*; (A) ITS region, (B) partial BT region, and (C) partial EF1-α region. Isolate sequences from this study, marked with black arrows, were compared to various *Ophiostoma* spp. in the "*O. ulmi* complex".

Maximum likelihood values that exceed 0.65 are shown among the branches and are denoted by bold lines. GenBank accession numbers of previously published sequences are included.

The first group of *Ophiostoma* isolates (strains CGL101 to CGL113) was identified as *O. quercus* by phylogenetic analysis. The identity of this group could not be fully resolved to the species-level by analysis of the ITS barcode region, as it did not resolve separately from species from the "*O. ulmi* complex" (Fig 5A). Analysis of the partial BT gene region placed these isolates into two clades that grouped together with *O. quercus* isolates to form a single well-supported clade (Fig 5B). Similarly, analysis of the partial EF1-α gene region grouped all isolates with *O. quercus* to form a single well-supported clade (Fig 5C).

The second group of *Ophiostoma* isolates (strain CGL201) was identified by phylogenetic analysis as a putatively novel *Ophiostoma* sp. During analysis of the ITS barcode region, this group formed a single well-supported clade that was related to, but distinct from, *O. borealis*, *O. bacillisporum*, and *O. catonianum* (Fig 5A). Similarly, analysis of the partial BT gene region placed this group in a distinct and well-supported clade that was related to, but separate from, *O. catonianum* (Fig 5B). Phylogenetic analysis of the partial EF1-α gene region also placed this group in a single well-supported clade that was related to, but distinct from, *O. karelicum* (Fig 5C). The consistent, well-supported distinction of this group from other species in the "*O. ulmi* complex" strongly suggests the identification of an *Ophiostoma* species that has yet to be characterized.

Isolates from the genus *Cadophora* (strains CGL301 to CGL303) were identified as *C. spadicis*, based on comparison of ITS, EF1-α and BT sequences with those of closely-related *Cadophora* species [50] (Fig 6). DNA amplification of *Cadophora* isolates resulted in fragments of ~500bp for the ITS barcode region and ~500bp for the partial EF1-α gene region.

Phylogenetic analysis of the ITS barcode region grouped all isolates with *C. spadicis* to form a single well-supported clade (Fig 6A). Analyses of the partial EF1-α gene region and the partial BT gene region for *Cadophora* isolates produced similar results to align with *C. spadicis* (Fig 6B-6C).

Fig 6. Single-locus maximum likelihood phylogenetic trees showing the phylogenetic placement of *Cadophora* isolates associated with *Alniphagus aspericollis* (marked by black arrows); (A) ITS region, (B) partial EF1-α region, and (C) partial BT region. Maximum likelihood values which exceed 0.65 are shown among the branches and are indicated by bold lines. GenBank accession numbers of previously published sequences are included.

The sole isolate from the ophiostomatoid genus *Graphilbum* was identified as *G. fragrans* based upon the closest BLAST matches of its ITS barcode sequence (Table 1) and culture morphology (e.g., production of yellow pigmentation in MEA, as described by [65] and [66]) (Fig 6E). Single isolates of *Valsalnicola oxystoma* and *Chondrostereum purpureum* were also identified to the species-level by BLAST searches of their ITS barcode sequences (Table 1). Given that *G. fragrans*, *V. oxystoma* and *C. purpureum* were each only represented by one isolate, and no comprehensive genera-level phylogenetic studies are currently available for these species, no further phylogenetic analyses were conducted for these isolates.

Relative prevalence of filamentous fungus species

Neonectria sp. nov., *O. quercus, Ophiostoma* sp. nov., *C. spadicis*, and *Penicillium* spp. were each isolated from both adult beetles and gallery phloem samples (Table 2). Additionally,

Graphilbum fragrans, Beauveria spp., *Cladosporium* sp., *Acremonium* sp., and *Cosmospora* sp. were isolated only from adult beetles, while *Chondrostereum purpureum*, *Valsalnicola oxystoma*, *Pezicula* sp., and *Umbelopsis* sp. were isolated only from gallery phloem samples (Table 2).

Table 2. Overall prevalence of filamentous fungus species associated with *Alniphagus aspericollis* across seven sampling sites throughout the Greater Vancouver region (British Columbia, Canada).

**Fungus**	**Adult beetles**		**Gallery phloem**		**Concurrent isolations**		**Trees**	
	**n**	**%**	**n**	**%**	**n**	**%**	**n**	**%**
~~**Canker pathogens**~~								
Neonectria sp. nov.	36	66.7	32	59.3	29	53.7	17	94.4
**Ophiostomatoid fungi**								
Ophiostoma quercus	15	27.7	5	9.3	3	5.6	10	55.6
Ophiostoma sp. nov.	3	5.6	2	3.7	0	0.0	3	16.7
Graphilbum fragrans	1	1.9	0	0.0	0	0.0	1	5.6
**Other fungi**								
Cadophora spadicis	6	11.1	2	3.7	0	0.0	6	33.3
Beauveria spp.	3	5.6	0	0.0	0	0.0	2	11.1
Chondrostereum purpureum	0	0.0	1	1.9	0	0.0	1	5.6
Valsalnicola oxystoma	0	0.0	1	1.9	0	0.0	1	5.6

Penicillium spp.	12	22.2	4	7.4	1	1.9	7	38.9
Cladosporium sp.	2	3.7	0	0.0	0	0.0	2	11.1
Acremonium sp.	1	1.9	0	0.0	0	0.0	1	5.6
Cosmospora sp.	1	1.9	0	0.0	0	0.0	1	5.6
Pezicula sp.	0	0.0	1	1.9	0	0.0	1	5.6
Umbelopsis sp.	0	0.0	1	1.9	0	0.0	1	5.6
Unknown Ascomycota spp.	2	3.7	2	3.7	0	0.0	3	16.7

Frequency of isolation data are shown for adult beetles (n = 54), gallery phloem samples (n = 54), galleries (n = 54) with concurrent isolations from both adult beetles and phloem samples, and beetle-infested trees (n = 18) with isolations from either adult beetles or phloem samples.

Neonectria sp. nov. was the most frequently isolated filamentous fungus in our study, associated with approximately two-thirds of adult beetles and gallery phloem samples and almost all beetle-infested trees (Table 2). This canker fungus was concurrently isolated from both adult beetles and phloem samples collected from more than half of the sampled galleries. Aside from Pacific Spirit Regional Park, and Tynehead Regional Park in the case of phloem isolations, *N.* sp. nov. was isolated from more than 50% of adult beetles and gallery phloem samples collected at each study site (Table 3). Strikingly, *N.* sp. nov. was isolated from every beetle-infested tree at each site except Pacific Spirit Regional Park, where two out of the three sampled trees yielded *N.* sp. nov. isolates. At each site, however, isolation of *N.* sp. nov. never exceeded 90% for either adult beetle isolations or gallery phloem samples. *Neonectria* sp. nov. was only occasionally isolated from *A. aspericollis* larvae, pupae, and teneral adults.

Table 3. Prevalence of filamentous fungi associated with *Alniphagus aspericollis* including *Neonectria* sp. nov., *Ophiostoma quercus*, and *Ophiostoma* sp. nov. at each of seven sampling sites throughout the Greater Vancouver Region (British Columbia, Canada).

Fungus sp. (by site)	Adult beetles	Gallery phloem	Concurrent isolations	Trees	Larvae	Pupae	Teneral adults
***Neonectria* sp. nov.**							
Cypress Mountain	7 (9)	5 (9)	5 (9)	3 (3)	-	-	0 (1)
Pacific Spirit Regional Park	3 (9)	2 (9)	2 (9)	2 (3)	0 (1)	-	-
Tynehead Regional Park	5 (9)	4 (9)	3 (9)	3 (3)	0 (2)	1 (1)	-
Aldergrove Regional Park	7 (9)	6 (9)	6 (9)	3 (3)	1 (1)	2 (3)	0 (1)
Gates Park	5 (6)	4 (6)	4 (6)	2 (2)	0 (2)	-	1 (1)
Burnaby Lake Regional Park	2 (3)	3 (3)	2 (3)	1 (1)	-	-	-
Burnaby Mountain	7 (9)	8 (9)	7 (9)	3 (3)	1 (5)	-	-
Ophiostoma quercus							
Cypress Mountain	1 (9)	1 (9)	1 (9)	1 (3)	-	-	0 (1)
Pacific Spirit Regional Park	5 (9)	1 (9)	1 (9)	2 (3)	0 (1)	-	-
Tynehead Regional Park	3 (9)	2 (9)	1 (9)	2 (3)	1 (2)	0 (1)	-

Aldergrove Regional Park	0 (9)	0 (9)	0 (9)	0 (3)	0 (1)	0 (3)	0 (1)
Gates Park	2 (6)	1 (6)	0 (6)	2 (2)	0 (2)	-	0 (1)
Burnaby Lake Regional Park	1 (3)	0 (3)	0 (3)	1 (1)	-	-	-
Burnaby Mountain	3 (9)	0 (9)	0 (9)	2 (3)	0 (5)	-	-
***Ophiostoma* sp. nov.**							
Burnaby Mountain	3 (9)	2 (9)	0 (9)	3 (3)	0 (5)	-	-
Other sites	0 (45)	0 (45)	0 (45)	0 (15)	0 (6)	0 (4)	0 (3)

Frequency data are shown for isolations from adult beetles and gallery phloem samples, galleries with concurrent isolations from both adult beetles and phloem samples, beetle-infested trees with isolations from either adult beetles or phloem samples, and isolations from larvae, pupae and teneral adults. For each column category, values in parentheses refer to the total sample size per site.

Ophiostoma quercus was the second most frequently isolated filamentous fungus, but its association with *A. aspericollis* was much less consistent than *N.* sp. nov. Additionally, *O. quercus* was concurrently isolated from adult beetles and phloem samples from only 5.6% of sampled galleries (Table 2), nearly ten times lower than that for *N.* sp. nov. At five of seven study sites, *O. quercus* was consistently isolated from 33% of adult beetles, with the exceptions of Pacific Spirit Regional Park and Cypress Mountain where isolation rates were 56% and 11%, respectively (Table 3). Isolation frequency of *O. quercus* was even lower for gallery phloem samples from each site, with three sites yielding no isolates of *O. quercus* from phloem at all.

Beetle-infested trees surveyed at five of seven sites produced no isolates of *O. quercus*, and at Aldergrove Regional Park, *O. quercus* was not isolated from any beetle or gallery phloem sample. *Ophiostoma quercus* was almost never isolated from larvae, pupae, or teneral adults, the only exception being a single larva-derived isolate from Tynehead Regional Park.

Ophiostoma sp. nov. was rarely isolated, with an overall occurrence of just 3.7 – 16.7% on adult beetles, in galleries, and infested trees (Table 2). This was primarily because *O*. sp. nov. was only isolated from Burnaby Mountain, where 33% of adult beetles and 22% of gallery phloem samples yielded the fungus (Table 3). *Ophiostoma* sp. nov. was isolated from all 3 beetle-infested trees surveyed at Burnaby Mountain but not concurrently isolated from adult beetles and gallery phloem samples from the same gallery. This putatively novel species of *Ophiostoma* was never isolated from larvae, pupae, or teneral adults.

Cadophora spadicis and *Penicillium* spp. were the two most prominent filamentous fungal taxa that did not fall into canker pathogen or ophiostomatoid fungus categories. *Cadophora spadicis* was the third most frequently isolated filamentous fungus species, yet it was never concurrently isolated from beetles and gallery phloem samples from the same gallery (Table 2). *Penicillium* spp., comprising up to 10 different species in our study, had an overall occurrence of 7.4 – 38.9% (Tables 1 and 2). All other observed filamentous fungus species were rarely isolated. Except *Beauveria* spp., which occurred on 5.6% of adult beetles, these fungi were each isolated from only one or two adult beetle or gallery phloem samples (Table 2).

Discussion

We found the alder bark beetle, *A. aspericollis*, to be associated with a diverse assemblage of filamentous fungus species that differed markedly in composition when compared

with conifer tree-killing bark beetles [5,20,28]. Surprisingly, the most frequently isolated fungus from *A. aspericollis*, gallery phloem, and nearly all beetle-infested trees was *Neonectria* sp. nov., a heretofore unknown type of ascomycete bark beetle associate that is most closely related to *N. major* (Wollenw.) Castl. & Rossman (Hypocreales: Nectriaceae). *Neonectria* fungi are well-known canker pathogens of trees; *N. major* is specific to the host genus *Alnus* [47], and forms perennial target-shaped stem cankers that eventually lead to limb damage and dieback over several years [40,67]. *Neonectria major* has previously been reported from red alder stands in the Pacific Northwest, as well as from *Alnus* spp. in Norway and France [40,45,47,67], and has even been utilized as a biological control agent for red alder within conifer plantations in British Columbia [68,69]. *Neonectria* fungi do not possess sticky conidia or ascospores such as those of ophiostomatoid entomochoric fungi, and like *Geosmithia* associates of bark beetles, do not appear to be specifically adapted for insect dispersal [12,27,47,70].

Ophiostoma quercus (Georgev.) Nannf. (Ophiostomatales: Ophiostomataceae) was the second most frequently isolated fungal associate of *A. aspericollis*, yet it was infrequently isolated from adult beetles and even more rarely cultured from gallery phloem. *Ophiostoma quercus* is a sapwood-staining ophiostomatoid fungus that occurs worldwide in a variety of hardwoods genera (e.g., *Quercus, Fagus, Acer, Betula, Eucalyptus, Castanea*) [60] and is considered to be saprotrophic or mildly pathogenic [71,72]. *Ophiostoma quercus* has been found on *Alnus incana* in Latvia [73], but our study is the first to report *O. quercus* from *A. rubra*. As a casual associate of many hardwood-invading bark beetles worldwide [74,75], it was probable that *O. quercus* would be occasionally isolated from *A. aspericollis*. Our study also reports the discovery of a new ophiostomatoid species, *Ophiostoma* sp. nov., a minor associate of *A. aspericollis*. *Ophiostoma* sp. nov. was only found at one of seven sampling sites, Burnaby

Mountain, where it was isolated slightly more frequently than *O. quercus* from all beetle-infested trees but only occasionally cultured from adult *A. aspericollis* and gallery phloem. Many bark beetle species have more than one ophiostomatoid fungal partner [75-77], and the relative dominance of different ophiostomatoid partners may vary with geographic location and environmental conditions [27,78].

Other tree pathogens and decay fungi, including *C. spadicis* and *V. oxystoma*, as well as various ubiquitous saprobes and the entomopathogenic *Beauveria* spp., were identified on *A. aspericollis* and in gallery phloem. These fungi were rarely isolated, however, and were obtained from either adult beetles or gallery phloem samples, but not both. For instance, *Beauveria* spp. was isolated from adult beetles only, which is consistent with the entomopathogenic function of these fungi [79,80]. Likewise, fungi such as *V. oxystoma* and *C. purpureum* were only isolated from gallery phloem samples, which is consistent with their ecological role as tree pathogens and decay fungi [81,82].

This study represents the first record of *C. spadicis* (Prodi, Sandalo, Tonti, Nipoti & A. Pisi) Travadon, Lawrence, Rooney-Latham, Gubler, Wilcox, Rolshausen & K. Baumgartner (Order: Helotiales) ~~on red alder,~~ and to the best of our knowledge, in western Canada. The genus *Cadophora* comprises various plant pathogens and decay fungi [83,84] that are not known to be vectored by insects. *Cadophora spadicis* has been reported as a decay agent of grapevine, *Vitis* spp., in North America, and is known to induce trunk hypertrophy in kiwifruit, *Actinia deliciosa*, in Italy [50,53]. Other *Cadophora* species have been occasionally isolated from *Alnus incana* and *A. glutinosa* [73,81]. In this study, *C. spadicis* was infrequently isolated from *A. aspericollis* and gallery phloem samples as it is probably a decay or pathogenic fungus of red alder that may occasionally utilize the entrance holes of *A. aspericollis* for access to host trees and may

therefore be coincidentally carried by the beetles. This passive association is also the likely case for other generalist saprophytes (e.g., *Penicillium* spp., *Cladosporium* sp.) that were isolated during this study. These ubiquitous wind-dispersed fungi have been found in similar frequencies in other bark beetle systems [76,85] and are not known to colonize living trees nor to be antagonists of bark beetles, unlike those reported by [86] and [87] (e.g., *Trichoderma harzianum*, *T. polysporum*, *Aspergillus fumigatus*, *A. nomius*). The ubiquitous saprophytic species identified by this study are therefore likely to be late colonizers of *A. aspericollis*-killed trees, or opportunistic hitchhikers of *A. aspericollis*, and are probably of little ecological importance to the beetle.

In keeping with our initial prediction, *A. aspericollis* did not display a consistent relationship with ophiostomatoid fungi but was instead casually associated with several species (i.e., *O. quercus*, *O.* sp. nov., and *G. fragrans*). Conifer-killing bark beetles are almost always closely associated with ophiostomatoid fungi, with the rare exception of a few resource-pulse driven species (e.g., *Tomicus piniperda*, *Hylurgus ligniperda*) [76,88]. Given that the host tree of *A. aspericollis*, *A. rubra*, fixes atmospheric nitrogen and its tissues are thereby rich in this limiting nutrient [37], selection for an association with ophiostomatoid fungi based upon their capacity to concentrate nitrogen in the phloem of their host trees [22,23] seems unlikely. The absence of an intimate association between *A. aspericollis* and ophiostomatoid fungi lends support to the assertion that the mutualistic relationship between conifer-killing bark beetles and ophiostomatoid fungi arose in part from the fungi’s capacity to improve the nutritional condition of phloem for developing beetles [21-23]. The loose association between *A. aspericollis* and ophiostomatoid fungi strongly suggests that these fungi do not provide any significant ecological benefits to *A. aspericollis*.

Neonectria sp. nov. was frequently isolated from both adult beetles and phloem within the same gallery, suggestive of a pathogen–vector relationship where *A. aspericollis* is capable of transmitting the fungus between host red alder trees. It is unclear, however, if *N*. sp. nov. has an obligate or facultative relationship with *A. aspericollis* since the fungus was not isolated at the high frequencies (i.e., 90-100%) observed in well-established bark beetle–fungus symbioses (e.g., *Dendroctonus rufipennis* and *Leptographium abietinum*, *Dendroctonus ponderosae* and *Ophiostoma* spp., *Scolytus scolytus* and *O. ulmi*) [89-91]. Little is known about the etiology of *N. major*, but reproductive spores of the closely-related *Neonectria ditissima* (Tul. & C. Tul.) Samuels and Rossman are usually wind or water-dispersed, gaining entry to host trees via stem wounds, bark cracks, and leaf scars [92]. Cootsona [67] suggested that drought stress predisposes red alder to *N. major* infection in the Pacific Northwest and did not discount the possibility that insects could vector *N. major.* Interestingly, we observed during sample collection that *N*. sp. nov. infection in *A. aspericollis*-infested red alder was not accompanied by typical *Neonectria* disease symptoms. Canker pathogens including *Neonectria* and *Geosmithia* species usually induce target-shaped cankers or prominent lesions to form on the limbs or stems as infections progress [67,92-94]. None of the red alders sampled during this study displayed prominent canker or lesion symptoms on their main stems, raising the possibility that *N*. sp. nov. may proliferate throughout tree tissues in a different manner when transmitted by *A. aspericollis* than when the pathogen infects a host on its own.

Hardwood-infesting bark beetles maintain relationships of varying degrees of intimacy with both ophiostomatoid fungal associates and non-ophiostomatoid fungi including canker pathogens from the order Hypocreales, which includes *Neonectria* and *Geosmithia*. *Scolytus scolytus* nearly always carries *O. ulmi* as part of the Dutch elm disease complex [89], while *S.*

multistriatus and *S. schevyrewi*, two less efficient vectors of Dutch elm disease, are associated with *O. novo-ulmi* at inconsistent frequencies (i.e., 9-92%) [95]. The birch bark beetle *S. ratzeburgi* is consistently associated with *O. karelicum*, a moderately pathogenic fungus that likely has similar etiology to Dutch elm disease [59,96]. *Scolytus intricatus* and *Hylesinus varius*, which kill their respective weakened oak and ash hosts, are loosely and infrequently associated with ophiostomatoid fungi, including the non-virulent *O. quercus* [74,97]. *Scolytus intricatus* has formed a closer association with *Geosmithia pallida*, the non-ophiostomatoid causal agent of foamy bark canker in coast live oak that is also vectored by *Pseudopityophthorus pubipennis* [70,93,94,97]. *Pseudopityophthorus* spp. are known to transmit oak wilt, *B. fagacearum* [32,98]. The walnut twig beetle, *Pityophthorus juglandis*, vectors the phytopathogen *Geosmithia morbida*, a beetle–fungus complex responsible for the thousand canker disease of black walnut in the western United States [93]. *Ceratocystis* spp. thought to be vectored by the mango bark beetle, *Hypocryphalus mangiferae*, cause diseases of mango trees in plantations worldwide [99-101].

Future research should focus on clarifying the ecological relationship between *A. aspericollis* and its *N.* sp. nov. partner, specifically whether this association is strictly casual or representative of a symbiosis that is mutualistic, commensalistic, or antagonistic in nature. To determine whether *A. aspericollis* is a vector of *N.* sp. nov., experimental work should be directed toward addressing the criteria outlined by [102] for defining a pathogen–vector relationship. Further, assessing the effects of *N.* sp. nov. on the host colonization and reproductive success of *A. aspericollis* would reveal whether this potential symbiosis represents a mutualism. The *N.* sp. nov. isolates obtained during this study were nearly genetically identical, suggesting that this species originated from a single and recent evolutionary event that is worthy

of future investigation. Finally, additional work is required to morphologically describe the novel species of *Neonectria* and *Ophiostoma* identified by phylogenetics herein.

Acknowledgements

The authors thank members of the Forest Pathology Lab (Department of Forest and Conservation Sciences, UBC) including Hesther Yueh, Nicolas Feau, Dario Isidrio Ojeda, Padmini Herath, Sandra Cervantes, Monique Sakalidis, Angela Dale, Charlie Zha, Winnie Ho, and Barbara Wong, for their invaluable guidance; members of the Mycology Lab (Department of Botany, UBC) including Dr. Mary Berbee, Ludovic Le Renard, and Anna Bazzicalupo, for permission to use their equipment on occasion; and the Metro Vancouver Regional Parks Office, for permission to conduct field sampling. This research was funded by Natural Sciences and Engineering Research Council of Canada Discovery Grants to ALC (RGPIN-2015-04376) and RCH (RGPIN-2015-05279).

References

1. Bright DE Jr. The bark beetles of Canada and Alaska: Coleoptera: Scolytidae. Ottawa, Ontario: Canada Department of Agriculture, Biosystematics Research Institute, Research Branch; 1976. Publication 1576: 241 p.

2. Wood SL. The bark and ambrosia beetles of North and Central America (Coleoptera: Scolytidae), a taxonomic monograph. Great Basin Naturalist Memoirs No. 6. Provo, Utah: Brigham Young University; 1982. 135 p.

3. Wright LC, Berryman AA, Wickman BE. Abundance of the fir engraver, *Scolytus ventralis*, and the Douglas-fir beetle, *Dendroctonus pseudotsugae*, following tree defoliation by the Douglas-fir tussock moth, *Orgyia pseudotsugata*. Can Entomol. 1984; 116(3): 293-305. doi: 10.4039/Ent116293-3

4. Raffa KF, Phillips TW, Salom SM. Strategies and mechanisms of host colonization by bark beetles. In: Schowalter TD, Filip GM, editors. Beetle-pathogen interactions in conifer forests. London: Academic Press; 1993. pp. 102-128.

5. Paine TD, Raffa KF, Harrington TC. Interactions among scolytid bark beetles, their associated fungi, and live host conifers. Annu Rev Entomol. 1997; 42: 179-206.

6. Coulson RN. Population dynamics of bark beetles. Annu Rev Entomol. 1979; 24: 417-447. doi: 10.1146/annurev.en.24.010179.002221

7. Romme WH, Knight DH, Yavitt JB. Mountain pine beetle outbreaks in the Rocky Mountains: regulators of primary productivity? Am Nat. 1986; 127: 484-494. doi: 10.1086/284497

8. Baker WL, Veblen TT. Spruce beetles and fires in the nineteenth-century subalpine forests of western Colorado, USA. Arc Alp Res. 1990; 22: 65-80. doi: 10.1080/00040851.1990.12002766

9. Berg EE, Henry JD, Fastie CL, De Volder AD, Matsuoka SM. Spruce beetle outbreaks on the Kenai Peninsula, Alaska, and Kluane National Park and Reserve, Yukon Territory: relationship to summer temperatures and regional differences in disturbance regimes. For Ecol Manage. 2006; 227: 219-232. doi: 10.1016/j.foreco.2006.02.038

10. Kurz WA, Dymond CC, Stinson G, Rampley GJ, Neilson ET, Carroll AL, Ebata T, Safranyik L. Mountain pine beetle and forest carbon: feedback to climate change. Nature 2008; 452: 987-990. doi: 10.1038/nature06777

11. Raffa KF, Aukema BH, Bentz BJ, Carroll AL, Hicke JA, Turner MG, et al. Cross-scale drivers of natural disturbances prone to anthropogenic amplification: the dynamics of bark beetle eruptions. Bioscience. 2008; 58(6): 501-517. doi: 10.1641/B580607

12. Farrell BD, Sequeira AS, O'Meara BC, Normark BB, Chung JH, Jordal BH. The evolution of agriculture in beetles (Curculionidae: Scolytinae and Platypodinae). Evolution. 2001; 55(10): 2011-2027. doi: 10.1111/j.0014-3820.2001.tb01318.x

13. Barras SJ. Antagonism between *Dendroctonus frontalis* and the fungus *Ceratocystis minor*. Ann Entomol Soc Am. 1970; 63: 1187-1190. doi: 10.1093/aesa/63.4.1187

14. Bridges JR, Moser JC. Role of two phoretic mites in transmission of bluestain fungus, *Ceratocystis minor*. Ecol Entomol. 1983; 8: 9-12. doi: 10.1111/j.1365-2311.1983.tb00476.x

15. Klepzig KD, Moser JC, Lombadero FJ, Hofstetter RW, Ayres MP. Symbiosis and competition: complex interactions among beetles, fungi and mites. Symbiosis. 2001; 30: 83-96. Available from: https://www.fs.usda.gov/treesearch/pubs/2400

16. Whitney HS, Farris SH. Maxillary mycangium in the mountain pine beetle. Science 1970; 167: 54-55. doi: 10.1126/science.167.3914.54

17. Happ GM, Happ CM, Barras SJ. Fine structure of the prothoracic mycangium, a chamber for the culture of symbiotic fungi, in the southern pine beetle, *Dendroctonus frontalis*. Tissue Cell. 1971; 3: 295-308. doi: 10.1016/S0040-8166(71)80024-1

18. Six DL. Bark beetle-fungus symbioses. In: Bourtzis K, Miller T, editors. Insect Symbioses. Boca Raton: CRC press; 2003a. pp. 97-114. doi: 10.1201/9780203009918

19. Whitney HS. Relationships between bark beetles and symbiotic organisms. In: Mitton JB, Sturgeon KB, editors. Bark beetles in North American conifers. Austin: University of Texas Press; 1982. pp. 183-211.

20. Six DL, Wingfield MJ. The role of phytopathogenicity in bark beetle-fungus symbioses: a challenge to the classic paradigm. Annu Rev Entomol. 2011; 56: 255-72. Available from: http://hdl.handle.net/2263/15796

21. Six DL, Paine TD. Effects of mycangial fungi and host tree species on progeny survival and emergence of *Dendroctonus ponderosae* (Coleoptera: Scolytidae). Environ Entomol. 1998; 27(6): 1393-1401. doi: 10.1093/ee/27.6.1393

22. Ayres MP, Wilkens RT, Ruel JJ, Lombardero MJ, Vallery E. Nitrogen budgets of phloem-feeding bark beetles with and without symbiotic fungi. Ecology. 2000; 81(8): 2198-2210. doi: 10.1890/0012-9658(2000)081[2198:NBOPFB]2.0.CO;2

23. Bleiker KP, Six DL. Dietary benefits of fungal associates to an eruptive herbivore: potential implications of multiple associates on host population dynamics. Environ Entomol. 2007; 36(6): 1384-1396. doi: 10.1093/ee/36.6.1384

24. Lieutier F, Yart A, Salle A. Stimulation of tree defenses by ophiostomatoid fungi can explain attack success of bark beetles in conifers. Ann For Sci. 2009; 66(8): 801. doi: 10.1051/forest/2009066

25. Kirisits T, Offenthaller I. Xylem sap flow of Norway spruce after inoculation with the blue-stain fungus *Ceratocystis polonica*. Plant Pathol. 2002; 51(3): 359-364. doi: 10.1046/j.1365-3059.2002.00722.x

26. Urban J, Dvořák M. Sap flow-based quantitative indication of progression of Dutch elm disease after inoculation with *Ophiostoma novo-ulmi*. Trees. 2014; 28(6): 1599-1605. doi: 10.1007/s00468-014-1068-0

27. Klepzig KD, Six DL. Bark beetle–fungal symbiosis: context dependency in complex associations. Symbiosis. 2004; 37: 189-205.

28. Six DL. Ecological and evolutionary determinants of bark beetle–fungus symbioses. Insects. 2012; 3: 339-366. doi: 10.3390/insects3010339

29. de Beer ZW, Seifert KA, Wingfield MJ. A nomenclator for ophiostomatoid genera and species in the Ophiostomatales and Microascales. CBS Biodivers Ser. 2013; 12: 245-322. Available from: https://www.fungaltaxonomy.org/files/6914/0613/2818/deBeeretal.2013.OphioNomenclator.pdf

30. Ploetz RC, Hulcr J, Wingfield MJ, de Beer ZW. Destructive tree diseases associated with ambrosia and bark beetles: black swan events in tree pathology? Plant Dis. 2013; 95(7): 856-872. doi: 10.1094/PDIS-01-13-0056-FE

31. Ohmart CP. Why are there so few tree-killing bark beetles associated with angiosperms? Oikos. 1989; 54(2): 242-245. doi: 10.2307/3565273

32. Rexrode CO, Jones TW. Oak bark beetles – important vectors of oak wilt. J Forest. 1970; 68(5): 294-297. doi: 10.1093/jof/68.5.294

33. Webber JF, Brasier CM. The transmission of Dutch elm disease: a study of the process involved. In: Anderson JM, Rayner ADM, Walton DWH, editors. Invertebrate-microbial interactions: joint symposium of the British Mycological Society and the British Ecological Society. University of Exeter, Exeter, United Kingdom, September 1982: Cambridge University Press, Cambridge, United Kingdom; 1984. pp. 271-306.

34. Santini A, Faccoli M. Dutch elm disease and elm bark beetles: a century of association. Iforest. 2015; 8: 126-134. doi: 10.3832/ifor1231-008

35. Chamberlin WJ. The Scolytoidea of the Northwest. Corvallis: Oregon State University Press; 1958. Available from: https://ir.library.oregonstate.edu/concern/technical_reports/st74cw353

36. Borden JH. Observations on the life history and habits of *Alniphagus aspericollis* (Coleoptera: Scolytidae) in southwestern British Columbia. Can Entomol. 1969; 101(8): 870-878. doi: 10.4039/Ent101870-8

37. Torrey JG. Nitrogen fixation by actinomycete-nodulated angiosperms. BioScience 1978; 28: 586–592. doi: 10.2307/1307515

38. Harrington CA. *Alnus rubra* Bong. Red alder. In: Burns RM, Honkala BH, editors. Silvics of North America, vol. 2, hardwoods. Washington, DC: USDA Forest Service; 1990. pp. 116-123.

39. Sieber TN, Sieber-Canavesi F, Dorworth CE. Endophytic fungi of red alder (*Alnus rubra*) leaves and twigs in British Columbia. Can J Bot. 1991; 69: 407-411. doi: 10.1139/b91-056

40. Sims L, Goheen E, Kanaskie A, Hansen E. Alder canopy dieback and damage in western Oregon riparian ecosystems. Northwest Sci. 2015; 89(1): 34-46. doi: 10.3955/046.089.0103

41. Schoch CL, Seifert KA, Huhndorf S, Robert V, Spouge JL, Levesque CA, et al. Nuclear ribosomal internal transcribed spacer (ITS) region as a universal DNA barcode marker for Fungi. Proc Natl Acad Sci USA. 2012; 109(16): 6241-6246. doi: 10.1073/pnas.1117018109

42. White TJ, Bruns TD, Lee SB, Taylor JW. Amplification and direct sequencing of fungal ribosomal RNA genes for phylogenetics. In: Innis MA, Gelfand DH, Sninsky JJ, White TJ, editors. PCR Protocols: a Guide to Methods and Applications. San Diego: Academic Press, Inc.; 1990. pp. 315-322.

43. Gardes M, Bruns TD. ITS primers with enhanced specificity for basidiomycetes – application to the identification of mycorrhizae and rusts. Mol Ecol. 1993; 2: 113-118. doi: 10.1111/j.1365-294X.1993.tb00005.x

44. Liu YJ, Whelen S, Hall BD. Phylogenetic relationships among ascomycetes: evidence from an RNA polymerase II subunit. Mol Biol Evol. 1999; 16(12): 1799-1808. doi: 10.1093/oxfordjournals.molbev.a026092

45. Zhao P, Luo J, Zhuang WY, Liu XZ, Wu B. DNA barcoding of the fungal genus *Neonectria* and the discovery of two new species. Sci China Life Sci. 2011; 54(7): 664-674. doi: 10.1007/s11427-011-4184-8

46. Carbone I, Kohn LM. A method for designing primer sets for speciation studies in filamentous ascomycetes. Mycologia. 1999; 91(3): 553-556. doi: 10.1080/00275514.1999.12061051

47. Castlebury LA, Rossman AY, Hyten AS. Phylogenetic relationships of *Neonectria*/ *Cylindrocarpon* on Fagus in North America. Can J Bot. 2006; 84: 1417-1433. doi: 10.1139/b06-105

48. Glass NL, Donaldson GC. Development of primer sets designed for use with the PCR to amplify conserved genes from filamentous ascomycetes. Appl Environ Microbiol. 1995; 61(4): 1323-1330. doi: 10.1128/aem.61.4.1323-1330.1995

49. Alves A, Crous PW, Correia A, Phillips AJL. Morphological and molecular data reveal cryptic speciation in *Lasiodiplodia theobromae*. Fungal Divers. 2008; 28: 1-13.

50. Travadon R, Lawrence DP, Rooney-Latham S, Gubler WD, Wilcox WF, Rolshausen PE, et al. *Cadophora* species associated with wood-decay of grapevine in North America. Fungal Biol. 2015; 119: 53-66. doi: 10.1016/j.funbio.2014.11.002

51. Hall TA. BioEdit: a user-friendly biological sequence alignment editor and analysis program for Windows 95/98/NT. Nucleic Acids Symp Ser. 1999; 41: 95-98.

52. Guindon S, Dufayard JF, Lefort V, Anisimova M, Hordijk W, Gascuel O. New algorithms and methods to estimate maximum-likelihood phylogenies: assessing the performance of PhyML 3.0. Syst Biol. 2010; 59(3): 307-321. doi: 10.1093/sysbio/syq010

53. Prodi A, Sandalo S, Tonti S, Nipoti P, Pisi A. *Phialophora*-like fungi associated with kiwifruit elephantiasis. J Plant Pathol. 2008; 90: 487-494. Available from: https://www.jstor.org/stable/41998543

54. Summerbell RC, Gueidan C, Schroers H-J, de Hoog GS, Starink M, Rosete YA, et al. *Acremonium* phylogenetic overview and revision of *Gliomastix, Sarocladium,* and *Trichothecium*. Stud Mycol. 2011; 68: 139-162. doi: 10.3114/sim.2011.68.06

55. Herrera CS, Rossman AY, Samuels GJ, Pereira OL, Chaverri P. Systematics of the *Cosmospora viliuscula* species complex. Mycologia. 2015; 107(3): 532-557. doi: 10.3852/14-122

56. Cabral A, Groenewald JZ, Rego C, Oliveira H, Crous PW. *Cylindrocarpon* root rot: multi-gene analysis reveals novel species within the *Ilyonectria radicicola* species complex. Mycol Prog. 2012; 11: 655-688. doi: 10.1007/s11557-011-0777-7

57. de Beer ZW, Wingfield BD, Wingfield MJ. The *Ophiostoma piceae* complex in the Southern Hemisphere: a phylogenetic study. Mycol Res. 2003; 107(4): 469-476. doi: 10.1017/S0953756203007445

58. Zhou XD, de Beer ZW, Ahumada R, Wingfield BD, Wingfield MJ. *Ophiostoma* and *Ceratocystiopsis* spp. associated with two pine-infesting bark beetles in Chile. Fungal Divers. 2004; 15: 261-274.

59. Linnakoski R, de Beer ZW, Rousi M, Niemela P, Pappinen A, Wingfield MJ. Fungi, including *Ophiostoma karelicum* sp. nov., associated with *Scolytus ratzeburgi* infesting birch in Finland and Russia. Mycol Res. 2008; 112: 1475-1488. doi: 10.1016/j.mycres.2008.06.007

60. Grobbelaar JW, Aghayeva DN, de Beer ZW, Bloomer P, Wingfield MJ, Wingfield BD. Delimitation of *Ophiostoma quercus* and its synonyms using multiple gene phylogenies. Mycol Prog. 2009; 8: 221-236. doi: 10.1007/s11557-009-0594-4

61. Kamgan Nkuekam G, Solheim H, de Beer ZW, Grobbelaar JW, Jacobs K, Wingfield MJ, et al. *Ophiostoma* species, including *Ophiostoma borealis* sp. nov., infecting wounds of native broad-leaved trees in Norway. Cryptogam, Mycol. 2010; 31(3): 285-303. Available from: http://hdl.handle.net/2263/16486

62. Grobbelaar JW, de Beer ZW, Bloomer P, Wingfield MJ, Zhou XD, Wingfield BD. Discovery of *Ophiostoma tsotsi* on *Eucalyptus* wood chips in China. Mycoscience. 2011; 52: 111-118. doi: 10.1007/S10267-010-0081-4

63. Kamgan Nkuekam G, de Beer ZW, Wingfield MJ, Mohammed C, Carnegie AJ, Pegg GS, et al. *Ophiostoma* species (Ophiostomatales, Ascomycota), including two new taxa on eucalypts in Australia. Aust J Bot. 2011; 59: 283-297. doi: 10.1071/BT10231

64. de Beer ZW, Wingfield MJ. Emerging lineages in the Ophiostomatales. In: Seifert KA, De Beer ZW, Wingfield MJ, editors. Ophiostomatoid fungi: expanding frontiers. CBS Biodivers Ser. 2013; 12: 21-46.

65. Jacobs K, Seifert KA. Fungi Canadenses No. 347. *Pesotum fragrans*. Can J Plant Pathol. 2004; 26: 79-80.

66. Paciura D, Zhou XD, De Beer ZW, Jacobs K, Ye H, Wingfield MJ. Characterisation of synnematous bark beetle-associated fungi from China, including *Graphium carbonarium* sp. nov. Fungal Divers. 2010; 40: 75-88. doi: 10.1007/s13225-009-0004-x

67. Cootsona C. Identification and distribution of *Neonectria major* causing cankers on red alder (*Alnus rubra*). M.Sc. Thesis, College of Forest Resources, University of Washington. 2006.

68. Dorworth CE. Biological control of red alder (*Alnus rubra*) with the fungus *Nectria ditissima*. Weed Technol. 1995; 9(2): 243-248. doi: 10.1017/S0890037X00023289

69. Dorworth CE, Macey DE, Sieber TN, Woods TAD. Biological control of red alder (*Alnus rubra*) with indigenous pathogenic Ascomycotina. Can J Plant Pathol. 1996; 18(4): 315-324. doi: 10.1080/07060669609500582

70. Kolařík M, Kubátová A, Hulcr J, Pažoutová S. *Geosmithia* fungi are highly diverse and consistent bark beetle associates: evidence from their community structure in temperate Europe. Microb Ecol. 2008; 55: 65-80. doi: 10.1007/s00248-007-9251-0

71. Heath RN, Van der Line M, Groeneveld H, Wingfield BD, Wingfield MJ, Roux J. Factors influencing infection of *Acacia mearnsii* by the wilt pathogen *Ceratocystis albifundus* in South Africa. For Pathol. 2010; 40: 500-509. doi: 10.1111/j.1439-0329.2009.00626.x

72. Selochnik NN, Pashenova NV, Sidorov E, Wingfield MJ, Linnakoski R. Ophiostomatoid fungi and their roles in *Quercus robur* die-back in Tellermann forest, Russia. Silva Fenn. 2015; 49(5): 1-16. doi: 10.14214/sf.1328

73. Arhipova N, Gaitnieks T, Donis J, Stenlid J, Vasaitis R. Decay, yield loss and associated fungi in stands of grey alder (*Alnus incana*) in Latvia. Forestry. 2011; 84(4): 337-348. doi: 10.1093/forestry/cpr018

74. Kirisits T. Fungal associates of European bark beetles with a special emphasis on the ophiostomatoid fungi. In: Lieutier F, Day KR, Battisti A, Grégoire J, Evans HF, editors.

Bark and Wood Boring Insects in Living Trees in Europe, a Synthesis. Dordrecht: Springer Netherlands; 2007. pp. 181-236. doi: 10.1007/978-1-4020-2241-8_10

75. Linnakoski R, de Beer ZW, Rousi M, Solheim H, Wingfield MJ. *Ophiostoma denticiliatum* sp. nov. and other *Ophiostoma* species associated with the birch bark beetle in southern Norway. Persoonia. 2009; 23: 9-15. doi: 10.3767/003158509X468038

76. Jankowiak R. Fungi associated with *Tomicus piniperda* in Poland and assessment of their virulence using Scots pine seedlings. Ann For Sci. 2006; 63: 801-808. doi: 10.1051/forest:2006063

77. Yamaoka Y, Masuya H, Ohtaka N, Goto H, Kaneko S, Kuroda Y. *Ophiostoma* species associated with bark beetles infesting three *Abies* species in Nikko, Japan. J For Res. 2004; 9: 67-74. doi: 10.1007/s10310-003-0056-9

78. Roe AD, James PMA, Rice AV, Cooke JEK, Sperling FAH. Spatial community structure of mountain pine beetle fungal symbionts across a latitudinal gradient. Microb Ecol. 2011; 62: 347-360. doi: 10.1007/s00248-011-9841-8

79. Harrar JG, Ellis RP. The biology of a species of *Beauveria* from the southern pine bark beetle. Proc W Va Acad Sci. 1940; 1: 211.

80. Doberski JW. Comparative laboratory studies on three fungal pathogens of the elm bark beetle *Scolytus scolytus*: pathogenicity of *Beauveria bassiana, Metarhizium anisopliae,* and *Paecilomyces farinosus* to larvae and adults of *S. scolytus*. J Invertebr Pathol. 1981; 37(2): 188-194. doi: 10.1016/0022-2011(81)90074-4

81. Arhipova N, Gaitnieks T, Donis J, Stenlid J, Vasaitis R. Heart-rot and associated fungi in *Alnus glutinosa* stands in Latvia. Scand J Forest Res. 2012; 27(4): 327-336. doi: 10.1080/02827581.2012.670727

82. Walker DM, Rossman AY. *Valsalnicola oxystoma* (Rehm) D.M. Walker & Rossman, *comb. nov*. Fungal Planet. 2012; 128: 149. doi: 10.3767/003158512X661589

83. Allington WB, Chamberlain DW. Brown stem rot of soybean. Phytopathology. 1948; 38: 793-802.

84. Di Marco S, Calzarano F, Osti F, Mazzullo A. Pathogenicity of fungi associated with a decay of kiwifruit. Australas Plant Pathol. 2004; 33: 337-342. doi: 10.1071/AP04024

85. Safranyik L, Whitney HS, Bleiker KP. A survey of microorganisms from the spruce beetle in central British Columbia. Information Report. Victoria: Pacific Forestry Centre, Canadian Forest Service; 2010. Report No.: BC-X-420.

86. Jassim HK, Foster HA, Fairhurst CP. Biological control of Dutch elm disease: larvicidal activity of *Trichoderma harzianum*, *T. polysporum* and *Scytalidium lignicola* in *Scolytus scolytus* and *S. multistriatus* reared in artificial culture. Ann Appl Biol. 1990; 117: 187-196. doi: 10.1111/j.1744-7348.1990.tb04206.x

87. Cardoza YJ, Klepzig KD, Raffa KF. Bacteria in oral secretions of an endophytic insect inhibit antagonistic fungi. Ecol Entomol. 2006; 31: 636-645. doi: 10.1111/j.1365-2311.2006.00829.x

88. Davydenko K, Vasaitis R, Meshkova V, Menkis A. Fungi associated with the red-haired bark beetle, *Hylurgus ligniperda* (Coleoptera: Curculionidae) in the forest-steppe zone in eastern Ukraine. Eur J Entomol. 2014; 111(4): 561-565. doi: 10.14411/eje.2014.070

89. Webber JF. Relative effectiveness of *Scolytus scolytus*, *S. multistriatus* and *S. kirschi* as vectors of Dutch elm disease. Eur J Forest Pathol. 1990; 20: 184-192. doi: 10.1111/j.1439-0329.1990.tb01129.x

90. Six DL. A comparison of mycangial and phoretic fungi of individual mountain pine beetles. Can J For Res. 2003b; 33: 1331-1334. doi: 10.1139/x03-047

91. Six DL, Bentz BJ. Fungi associated with the North American spruce beetle, *Dendroctonus rufipennis*. Can J For Res. 2003; 33: 1815-1820. doi: 10.1139/x03-107

92. Weber RWS. Biology and control of the apple canker fungus *Neonectria ditissima* (syn. *N. galligena*) from a Northwestern European perspective. Erwerbs-Obstbau. 2014; 56: 95-107. doi: 10.1007/s10341-014-0210-x

93. Kolařík M, Freeland E, Utley C, Tisserat N. *Geosmithia morbida* sp. nov., a new phytopathogenic species living in symbiosis with the walnut twig beetle (*Pityophthorus juglandis*) on *Juglans* in USA. Mycologia. 2011; 103(2): 325-332. doi: 10.3852/10-124

94. Lynch SC, Wang DH, Mayorquin JS, Rugman-Jones PF, Stouthamer R, Eskalen A. 2014. First report of *Geosmithia pallida* causing foamy bark canker, a new disease on coast live oak (*Quercus agrifolia*), in association with *Pseudopityophthorus pubipennis* in California. Plant Dis. 2014; 98(9): 1276. doi: 10.1094/PDIS-03-14-0273-PDN

95. Jacobi WR, Koski RD, Harrington TC, Witcosky JJ. Association of *Ophiostoma novo-ulmi* with *Scolytus schevyrewi* (Scolytidae) in Colorado. Plant Dis. 2007; 91: 245-247. doi: 10.1094/PDIS-91-3-0245

96. Jankowiak R. First report of *Ophiostoma karelicum* from birch stands in Poland. Phytopathologia. 2011; 59: 55-58. Available from: https://www.cabi.org/isc/FullTextPDF/2011/20113270897.pdf

97. Šrůtka P. Transmission of fungus spores by bark beetles of the genus *Scolytus* with respect to the oak bark beetle (*Scolytus intricatus* Ratz.). Lesnictví-Forestry. 1996; 42(11): 510-517.

98. Buchanan WD. The small oak barkbeetle transmits the oak wilt disease. Journal of Forestry. 1958; 56: 414-415. doi: 10.1093/jof/56.6.414

99. Van Wyk M, Al Adawi AO, Khan IA, Deadman ML, Al Jahwari AA, Wingfield, BD, Ploetz R, Wingfield MJ. *Ceratocystis manginecans* sp. nov., causal agent of a destructive mango wilt disease in Oman and Pakistan. Fungal Divers. 2007; 27: 213-230.

100. Van Wyk M, Wingfield BD, Al-Adawi AO, Rossetto CJ, Ito MF, Wingfield MJ. Two new *Ceratocystis* species associated with mango disease in Brazil. Mycotaxon. 2011; 117: 381-404. doi: 10.5248/117.381

101. Al Adawi AO, Al Jabri RM, Deadman ML, Barnes I, Wingfield B, Wingfield MJ. The mango sudden decline pathogen, *Ceratocystis manginecans*, is vectored by *Hypocryphalus mangiferae* (Coleoptera: Scolytinae) in Oman. Eur J Plant Pathol. 2012; 135: 243–251. doi: 10.1007/s10658-012-0081-7

102. Leach JG. Insect transmission of plant diseases. New York: McGraw-Hill Book Co., Inc.; 1940.

Supplemental material

Table S1. **Strains of *Neonectria* sp. nov., *Ophiostoma quercus*, *Ophiostoma* sp. nov., and *Cadophora spadicis* isolated from *Alniphagus aspericollis* and gallery phloem, including their collection site(s) and GenBank accession numbers for barcode sequences (internal transcribed spacer (ITS) rDNA gene region, partial beta-tubulin (BT) gene, partial translation elongation factor 1-alpha (EF1-α) gene, and partial RNA polymerase II second largest subunit (RPB2) gene).** Mycelia from each isolate comprising the listed strains, with the exception of several *O. quercus* isolates that were not successfully sub-cultured after initial investigation, were preserved in 10% glycerol, flash-frozen in liquid nitrogen, and placed at -80°C for long-term storage in the Genomics and Forest Health (Hamelin) Lab at the University of British Columbia, Canada.

Species	Strain no.	Collection site(s)	GenBank accession No. (sequence ID)			
			ITS	BT	EF1-α	RPB2
Neonectria sp. nov.	CGL 401	All sites	(NEO01)	-	(NEOE1)	(NEOR1)

	CGL 402	GP	(NEO01)	-	(NEOE2)	(NEOR2)
Ophiostoma quercus	CGL 101	CM, PS, BM	(OPQ01)	(OPQB1)	(OPQE1)	-
	CGL 102	CM, TH, GP	(OPQ01)	(OPQB1)	(OPQE2)	-
	CGL 103	PS	(OPQ02)	(OPQB2)	(OPQE3)	-
	CGL 104	PS	(OPQ02)	(OPQB2)	(OPQE4)	-
	CGL 105	PS	(OPQ02)	(OPQB2)	(OPQE5)	-
	CGL 106	PS	(OPQ01)	(OPQB1)	(OPQE6)	-
	CGL 107	TH	(OPQ01)	(OPQB1)	(OPQE7)	-
	CGL 108	TH	(OPQ01)	(OPQB1)	(OPQE8)	-
	CGL 109	BL	(OPQ01)	(OPQB1)	(OPQE9)	-
	CGL 110	BM	(OPQ01)	(OPQB3)	(OPQE1)	-
	CGL 111	TH	(OPQ01)	(OPQB4)	(OPQE2)	-
	CGL 112	TH	(OPQ03)	(OPQB1)	(OPQE2)	-
	CGL 113	BL	(OPQ03)	(OPQB1)	(OPQE1)	-
Ophiostoma sp. nov.	CGL 201	BM	(OPS01)	(OPSB1)	(OPSE1)	-
Cadophora spadicis	CGL 301	AG, BM, CM	(CAD01)	(CADB1)	(CADE1)	-
	CGL 302	TH	(CAD02)	(CADB1)	(CADE1)	-
	CGL 303	AG	(CAD01)	(CADB2)	(CADE2)	-

Sampling site legend: CM = Cypress Mountain; PS = Pacific Spirit Regional Park; TH = Tynehead Regional Park; AG = Aldergrove Regional Park; GP = Gates Park; BL = Burnaby Lake Regional Park; BM = Burnaby Mountain Conservation Area.

Click here to access/download;Figure;Lee et al._Fig1.jpg

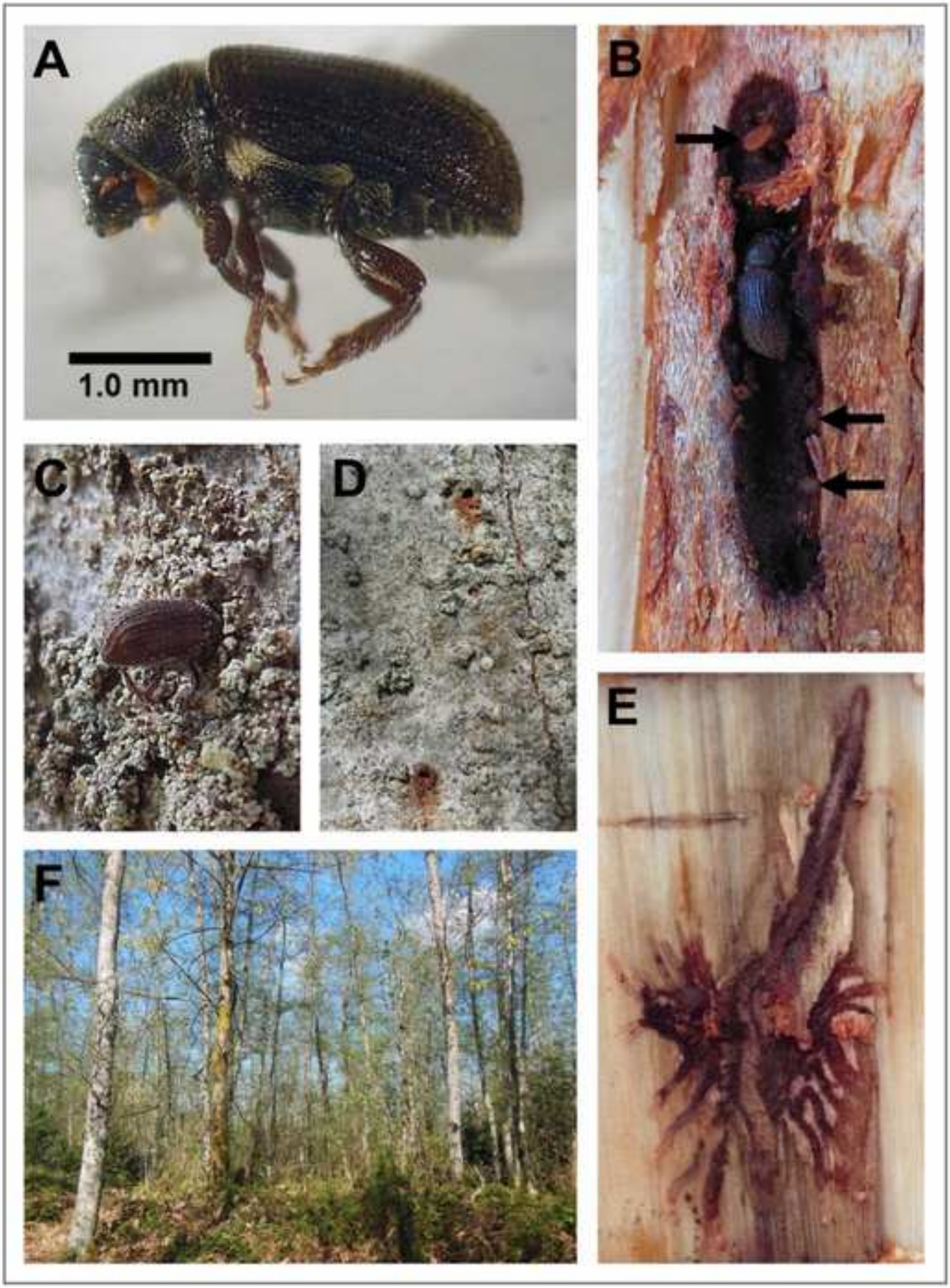

Figure 2

Click here to access/download;Figure;Lee et al._Fig2.jpg

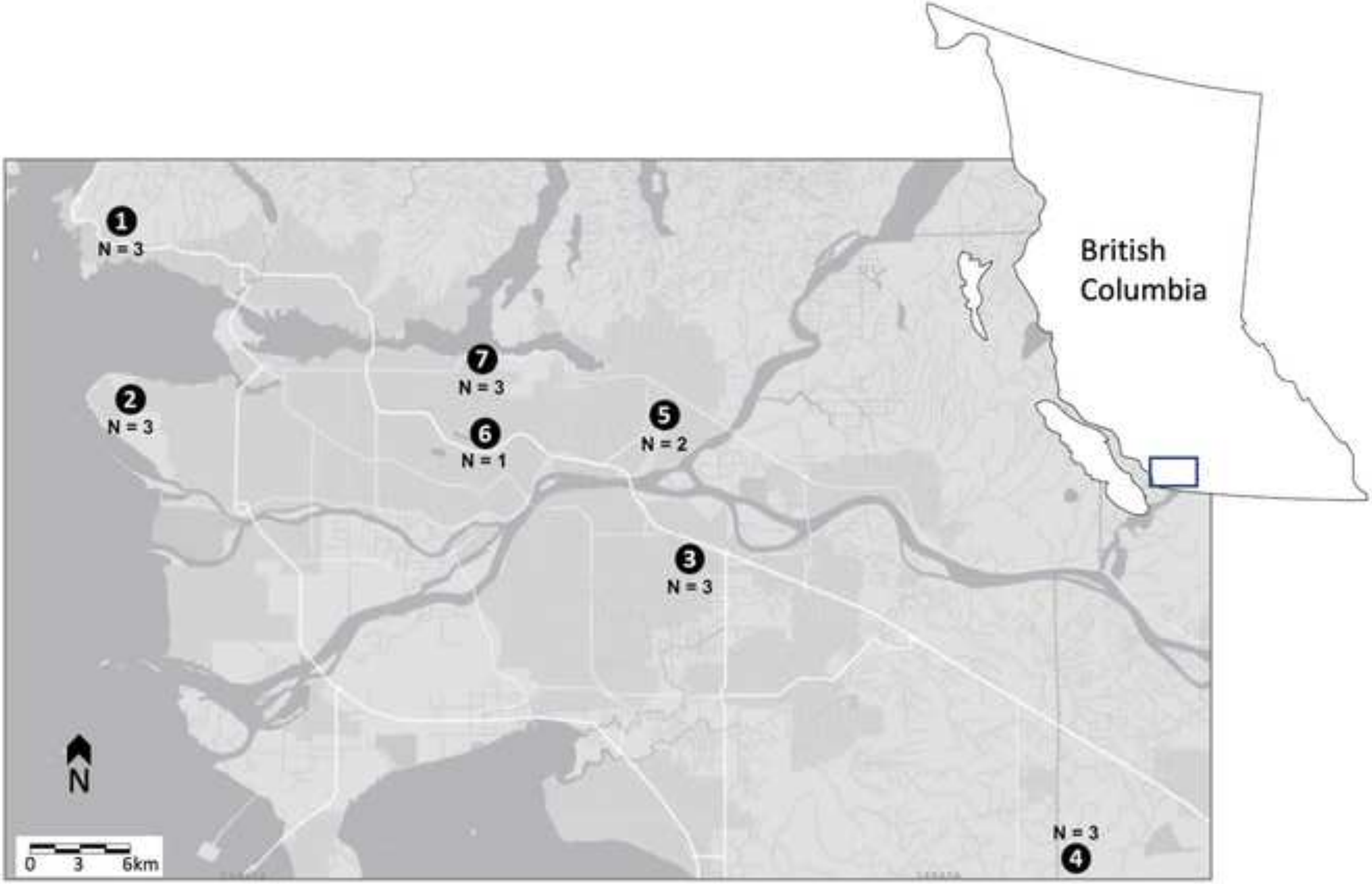

Figure 3

Click here to access/download;Figure;Lee et al._Fig3.jpg

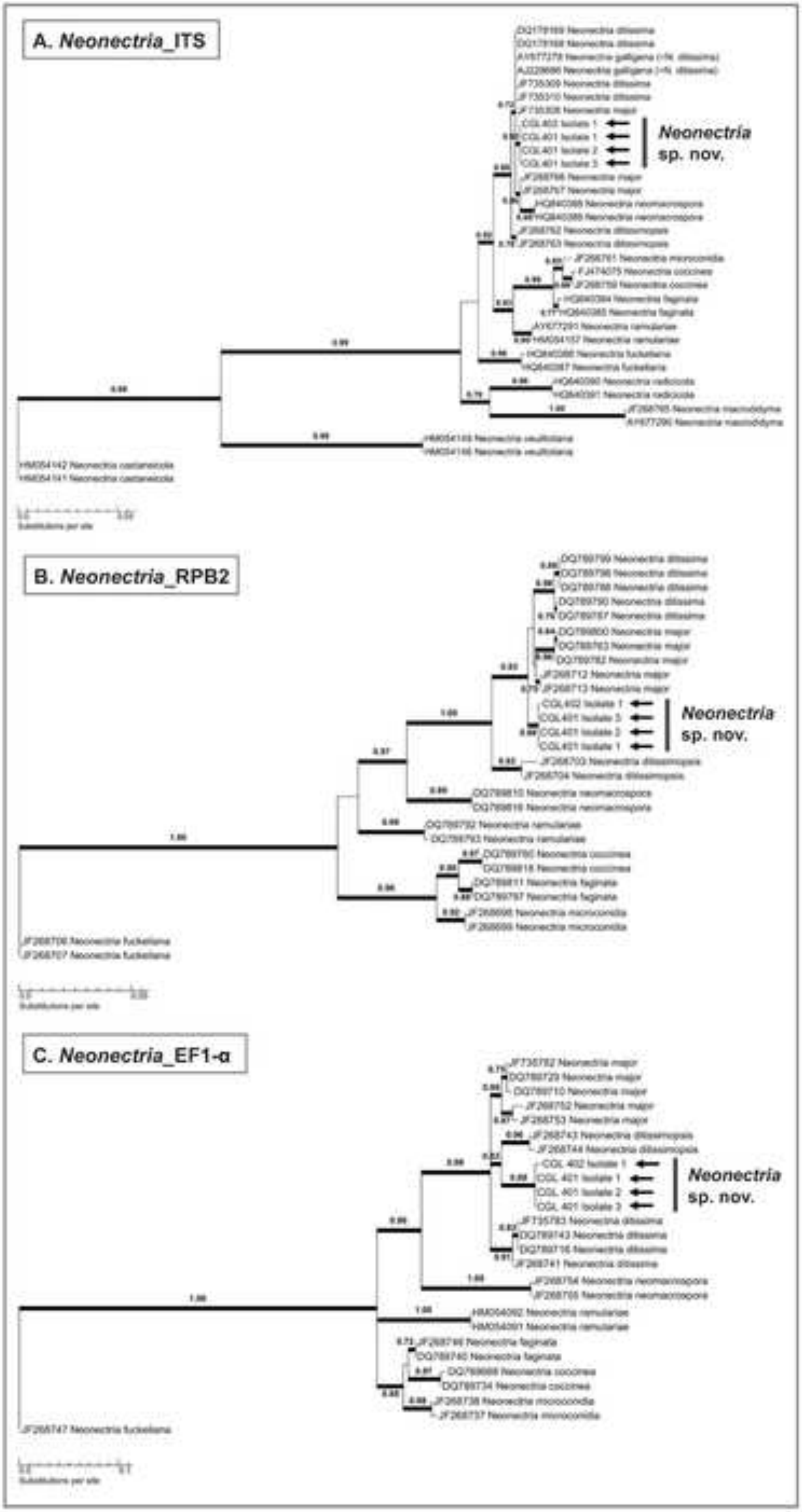

Click here to access/download;Figure;Lee et al._Fig4.jpg

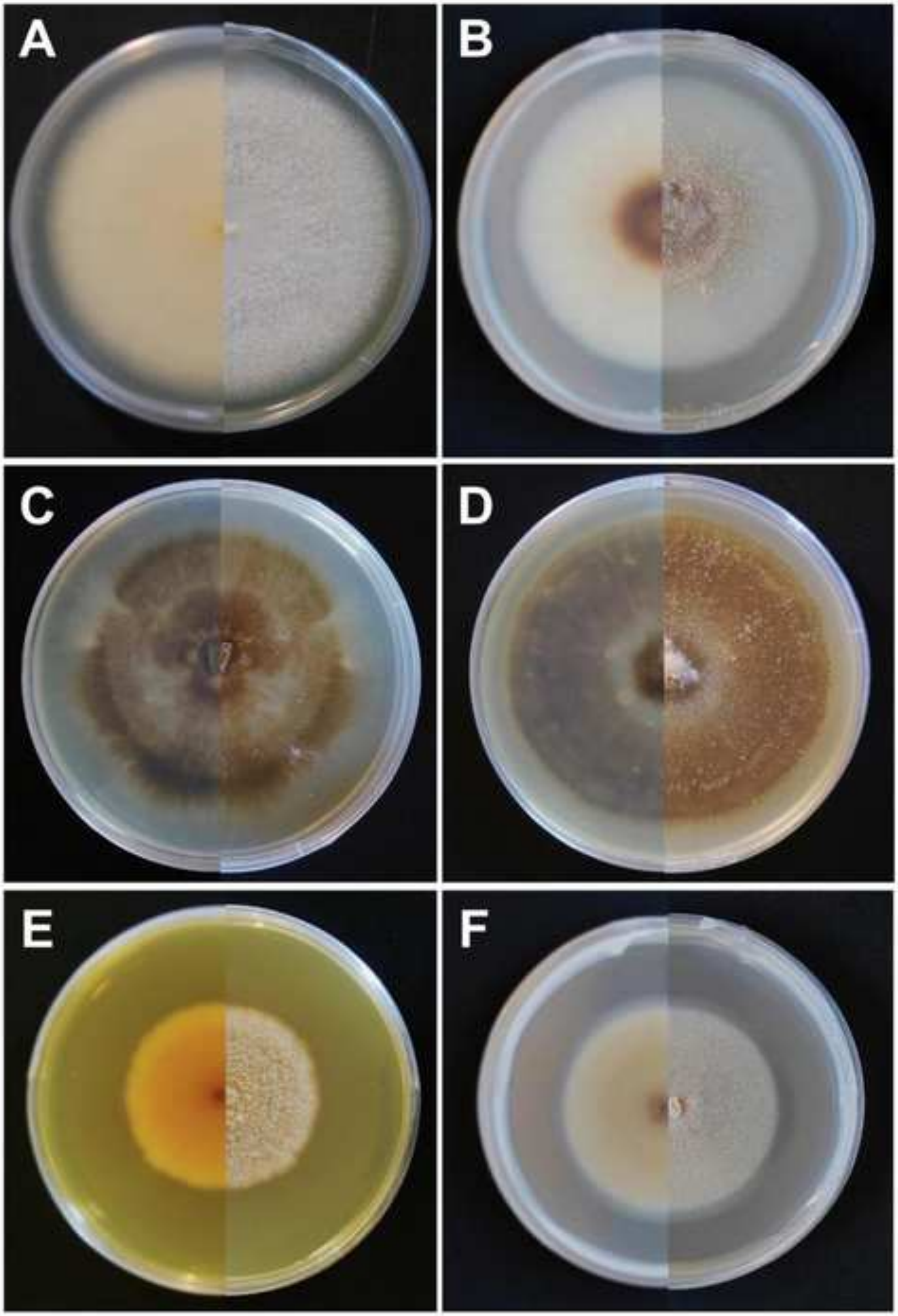

Figure 5

Click here to access/download;Figure;Lee et al._Fig5.jpg

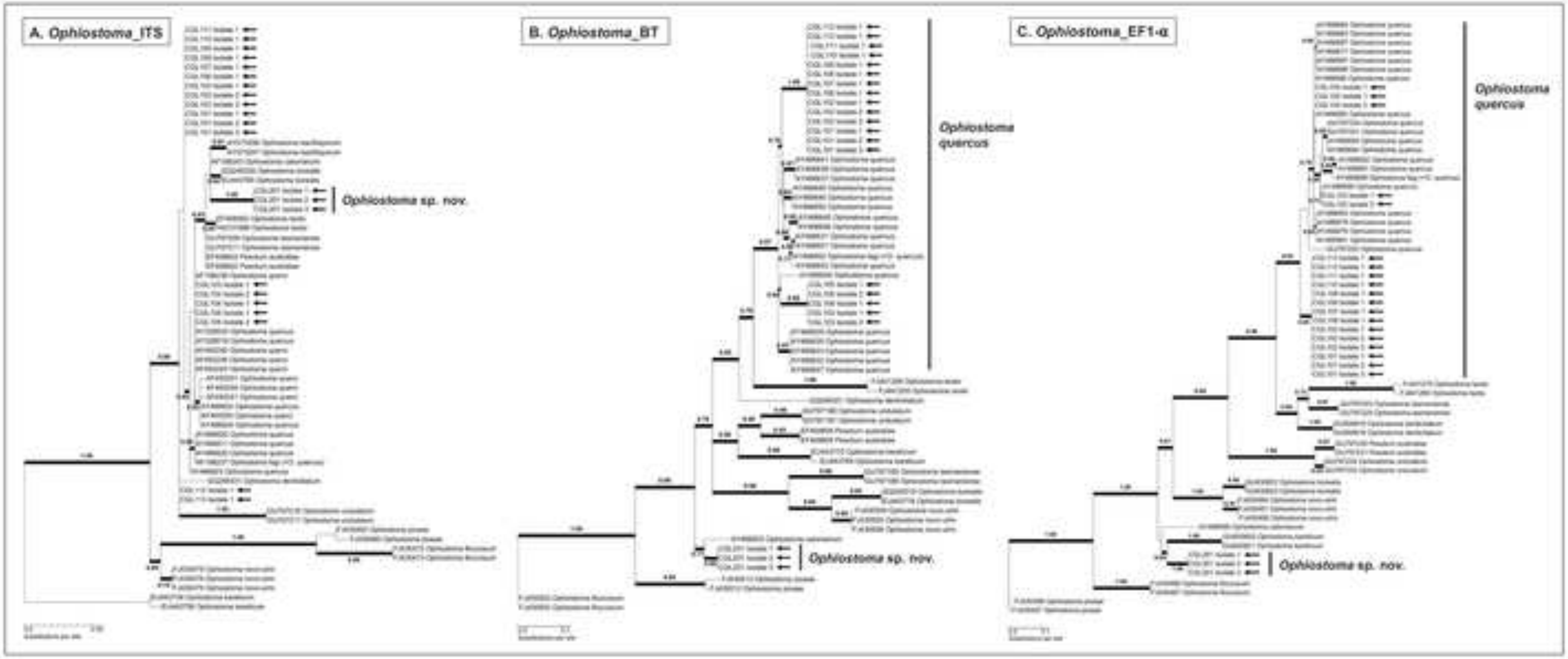

Click here to access/download;Figure;Lee et al._Fig6.jpg

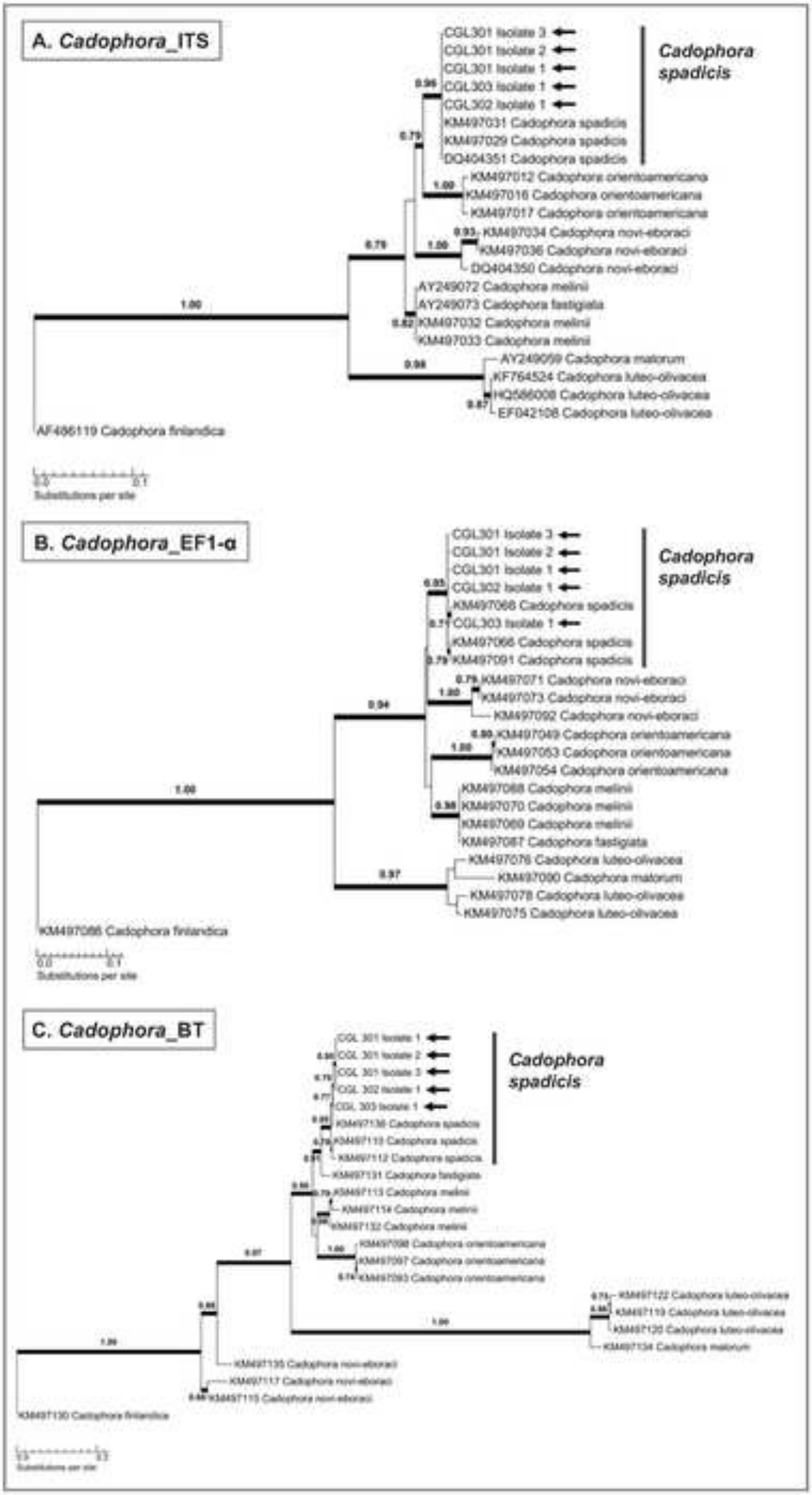

Supplement: Attachment — Submitted filename: PONE-D-22-28596_REV1.pdf [file pone.0284393.s002.pdf]
