# Supplementary material for: Filamentous fungal associates of the alder bark beetle, Alniphagus aspericollis, including an undescribed species of Neonectria
Source: PLoS One. 2023 May 8;18(5):e0284393. doi: 10.1371/journal.pone.0284393 (PMC10166519; doi:10.1371/journal.pone.0284393)
Supplement: Attachment — Submitted filename: Response to Reviewers_Lee et al_2023.docx [file pone.0284393.s003.docx]

10 March 2023

Dear Dr. Shamoun (Academic Editor, PLOS ONE),

We, Gervais Lee, Debra Wertman, Allan Carroll, and Richard Hamelin, the authors of “A novel type of bark beetle–fungus association between the hardwood-killing alder bark beetle, *Alniphagus aspericollis*, and a *Neonectria* canker pathogen” (Manuscript Number PONE-D-22-28596), thank the editors and reviewers for their constructive feedback on our article. This letter addresses each of the reviewers’ comments in turn, including those embedded in the PDF by the reviewers, indicating how suggestions were integrated to greatly improve our manuscript.

Reviewer comments are provided in italics with our responses follow each in plain text. Line numbers referenced by the Academic Editor and Reviewers correspond to the submitted manuscript, while the line numbers we reference correspond to the revised manuscript with track-changes.

**Academic Editor/Journal requests**

*1. Please ensure that your manuscript meets PLOS ONE's style requirements, including those for file naming. The PLOS ONE style templates can be found at*

*https://journals.plos.org/plosone/s/file?id=wjVg/PLOSOne_formatting_sample_main_body.pdf and https://journals.plos.org/plosone/s/file?id=ba62/PLOSOne_formatting_sample_title_authors_affiliations.pdf*

We have thoroughly reviewed the PLOS ONE style requirements and believe our manuscript is formatted accordingly.

*2. In your Methods section, please provide additional information regarding the permits you obtained for the work. Please ensure you have included the full name of the authority that approved the field site access and, if no permits were required, a brief statement explaining why.*

We have added a permitting statement to the methods (L109-111).

*3. Thank you for stating the following in the Acknowledgments Section of your manuscript:*

*“This research was funded by Natural Sciences and Engineering Research Council of Canada Discovery Grants to ALC (RGPIN-2015-04376) and RCH (RGPIN-2015-05279).”*

*We note that you have provided additional information within the Acknowledgements Section that is not currently declared in your Funding Statement. Please note that funding information should not appear in the Acknowledgments section or other areas of your manuscript. We will only publish funding information present in the Funding Statement section of the online submission form.*

*Please remove any funding-related text from the manuscript and let us know how you would like to update your Funding Statement. Currently, your Funding Statement reads as follows:*

*“ALC: RGPIN-2015-04376, Natural Sciences and Engineering Research Council of Canada, Discovery Grant, https://www.nserc-crsng.gc.ca/*

*RCH: RGPIN-2015-05279, Natural Sciences and Engineering Research Council of Canada, Discovery Grant, https://www.nserc-crsng.gc.ca/*

*The funding agency did not play any role in the study design, data collection and analysis, decision to publish, or preparation of the manuscript.”*

*Please include your amended statements within your cover letter; we will change the online submission form on your behalf.*

We have removed the funding information from the Acknowledgements Section and are happy with the funding statement as it is written above.

*4. In your Data Availability statement, you have not specified where the minimal data set underlying the results described in your manuscript can be found. PLOS defines a study's minimal data set as the underlying data used to reach the conclusions drawn in the manuscript and any additional data required to replicate the reported study findings in their entirety. All PLOS journals require that the minimal data set be made fully available. For more information about our data policy, please see http://journals.plos.org/plosone/s/data-availability.*

*Upon re-submitting your revised manuscript, please upload your study’s minimal underlying data set as either Supporting Information files or to a stable, public repository and include the relevant URLs, DOIs, or accession numbers within your revised cover letter. For a list of acceptable repositories, please see http://journals.plos.org/plosone/s/data-availability#loc-recommended-repositories. Any potentially identifying patient information must be fully anonymized.*

*We will update your Data Availability statement to reflect the information you provide in your cover letter.*

We have added text to our cover letter to address Data Availability and have added the recently issued GenBank accession numbers to the manuscript tables.

*5. We note that Figure 2 in your submission contain map images which may be copyrighted. All PLOS content is published under the Creative Commons Attribution License (CC BY 4.0), which means that the manuscript, images, and Supporting Information files will be freely available online, and any third party is permitted to access, download, copy, distribute, and use these materials in any way, even commercially, with proper attribution. For these reasons, we cannot publish previously copyrighted maps or satellite images created using proprietary data, such as Google software (Google Maps, Street View, and Earth). For more information, see our copyright guidelines: http://journals.plos.org/plosone/s/licenses-and-copyright.*

*We require you to either (1) present written permission from the copyright holder to publish these figures specifically under the CC BY 4.0 license, or (2) remove the figures from your submission:*

*2. If you are unable to obtain permission from the original copyright holder to publish these figures under the CC BY 4.0 license or if the copyright holder’s requirements are incompatible with the CC BY 4.0 license, please either i) remove the figure or ii) supply a replacement figure that complies with the CC BY 4.0 license. Please check copyright information on all replacement figures and update the figure caption with source information. If applicable, please specify in the figure caption text when a figure is similar but not identical to the original image and is therefore for illustrative purposes only.*

We have replaced the map in Figure 2 with one derived from the open source USGS National Map Viewer. We have revised the figure caption accordingly.

*6. If applicable, we recommend that you deposit your laboratory protocols in protocols.io to enhance the reproducibility of your results. Protocols.io assigns your protocol its own identifier (DOI) so that it can be cited independently in the future. For instructions see: https://journals.plos.org/plosone/s/submission-guidelines#loc-laboratory-protocols. Additionally, PLOS ONE offers an option for publishing peer-reviewed Lab Protocol articles, which describe protocols hosted on protocols.io. Read more information on sharing protocols at https://plos.org/protocols?utm_medium=editorial-email&utm_source=authorletters&utm_campaign=protocols.*

We have opted not to upload our protocols to protocols.io as we feel they are reproducible from the text in the Methods Section.

*7. While revising your submission, please upload your figure files to the Preflight Analysis and Conversion Engine (PACE) digital diagnostic tool, https://pacev2.apexcovantage.com/. PACE helps ensure that figures meet PLOS requirements. To use PACE, you must first register as a user. Registration is free. Then, login and navigate to the UPLOAD tab, where you will find detailed instructions on how to use the tool. If you encounter any issues or have any questions when using PACE, please email PLOS at figures@plos.org. Please note that Supporting Information files do not need this step.*

We have processed the figures using PACE and re-uploaded them with our submission.

**Reviewer #1 comments**

*The paper deals with the previously unknown association between a fungus and a bark beetle.*

*I read this paper with much interest and found it well written and generally satisfying. I think it deals with topics that, now, are of great interest to both entomologists and forest pathologists and therefore worth publishing.*

*However, I also found some minor inaccuracies that need to be resolved for a better overall result, and I also feel that the analysis of the literature pertaining to insect-fungal relationships should be expanded a bit so as not to come to hasty and inaccurate conclusions.*

*These general comments are valid throughout the manuscript:*

*1. In the title I would talk about fungi associated with* Alniphagus aspericollis *instead of* Neonectria *alone since the work done deals with several fungal species.*

We have updated the title of our manuscript to address this comment and additional issues raised below. The new title is: “Filamentous fungal associates of the alder bark beetle, *Alniphagus aspericollis*, including an undescribed species of *Neonectria*,” and the short title: “Fungal associates of the alder bark beetle include a species of *Neonectria.*”

*2. I would also not call the species of* Neonectria *that authors identified here a "canker pathogen" because it is not proven to be one: Koch's postulates have not been fulfilled yet. Belonging to a pathogen genus do not automatically mean that it is a pathogen. This is also the case of other fungi belonging to genera that usually contain pathogens, but they are not.* Ophiostoma quercus*, for example, share the same genus of some of the most infamous pathogens, but it is not a pathogen.*

This is a very important point raised by Reviewer #1 and we have updated the title, abstract, and main text of the manuscript to resolve the issue of *Neonectria* pathogenicity. It will be important for us to address Koch’s postulates in our forthcoming work on the alder bark beetle–*Neonectria* system as well, and this article will serve as a solid foundation for these studies. We concur that fungal pathogenicity cannot be assumed based on phylogenetic relationships and have revised our manuscript throughout to more accurately reflect what is known regarding the lifestyles of different taxa.

*3. In addition, the association the authors describe is not a “novel type of association”, but it is a “newly discovered” association.*

We agree with Reviewer #1 and have removed this phrasing from the title. See L547-549 of the Discussion Section for a more appropriate description of the alder bark beetle–*Neonectria* association.

*4. I would also avoid specifying in the title that the alder bark beetle is a hardwood-killing, because in many cases they kill plant already stressed by other factors that probably would have died anyway.*

While we have removed “hardwood-killing” from the title, we respectfully disagree with Reviewer #1 on this point as the alder bark beetle satisfies the definition of a pulse-driven irruptive bark beetle (Howe et al. 2022, Oecologia). The beetle attacks and eventually kills standing, live trees that may be healthy or stressed, and we have clarified this point on L89-90.

*5. So I suggest to change from “A novel type of bark beetle–fungus association between the hardwood-killing alder bark beetle,* Alniphagus aspericollis*, and a* Neonectria *canker pathogen” into: “A bark beetle-fungus association between the alder bark beetle,* Alniphagus aspericollis*, and a new species of* Neonectria*” or “Fungi associated to the alder bark beetle* Alniphagus aspericollis*”.*

We have updated the title accordingly, please see our response to Reviewer #1 point 1.

*Specific comments:*

*6. Regarding the Discussion chapter I would recommend to include not just cases of pathogens associated to insects, but also other guilds of fungi. In addition, if* Geosmithia morbida *is considered the agent of Thousand cankers disease,* G. pallida *is reported just in one case of being a pathogen (and not confirmed elsewhere), while in many other reports the ecological niche occupied by this fungus is not clear, but surely not a plant pathogen. See also the suggested literature. I think that the Discussion chapter should be reviewed in the light of an improved literature review.*

Our Discussion Section does review non-phytopathogenic fungal associates of bark beetles including saprotrophic/weakly pathogenic *Ophiostoma quercus* (L430-433), entomopathogenic *Beauveria* spp. (L465-467), Chondrostereum purpureum (L467-469) and *Cadophora* *spadicis* (L474-481) as decay agents, and saprotrophs including Penicillium spp., Cladosporium sp. (L481-532).

We have addressed the consistent associations between bark beetles and non-pathogenic Geosmithia spp., in contrast with *G. morbida*, on L423-427 and L608-611. Upon additional review of the literature, we do observe that *G. pallida* is likely not the causal agent of foamy bark canker and due to the ambiguity in this system we have removed this sentence and the corresponding article from our discussion – we thank Reviewer #1 for bringing this issue to our attention.

*PDF-embedded comments:*

*7. Ln 397: ~~on red alder~~ on* A. aspericollis

We have left this statement as written because *Cadophora spadicis* was isolated from gallery phloem as well as from adult beetles.

*8. Ln 459: It should be here mentioned that fungi of the genus* Geosmithia *are intimately associated to bark beetles and in most cases are not pathogens, as also reported in part of the cited literature see also Kolarik et al 2005 doi:10.1017/S0953756205003965; 2007 doi:10.1016/j.mycres.2007.06.010; and 2008 DOI: 10.1007/s00248-007-9251-0). In addition they are reported as ambrosia fungi (Kolarik and Kirkendall, 2010 doi:10.1016/j.funbio.2010.06.005) or mycoparasites (Pepori et al 2017 https://doi.org/10.1007/s00248-017-1062-3)*

Please see response to Reviewer #1 point 6 as we have updated the manuscript Discussion Section to address the non-phytopathogenic *Geosmithia* associates of bark beetles. We found the information needed for our updated statements on L423-427 and L608-611 in our existing citations (including Bettini et al. 2012 as suggested by Reviewer #2), but plan to incorporate more of the suggested literature on the lifestyles and habits of scolytine-associated *Geosmithia* fungi into upcoming manuscripts.

**Reviewer #2 comments**

*The manuscript titled “A novel type of bark beetle–fungus association between the hardwood-killing alder bark beetle,* Alniphagus aspericollis*, and a* Neonectria *canker pathogen” describes ID and occurrence of fungi associated with red alder and a bark beetle in the Vancouver, Canada area. The paper is clear, well written, thorough and the results support the conclusions.*

*1. While an analysis on community composition differences among beetles and phloem could have informed the discussion (e.g., contingency table chi-square and/or NMDS), I feel that the descriptive statistics and interpretation were clear.*

We appreciate this comment from Reviewer #2 and will consider community composition analyses for future studies.

*Specific comments:*

*2. Ln 95: Check journal style guide to check whether there should be a space between numbers and units. Here and throughout MS.*

It does appear that PLOS ONE articles contain spaces between numbers and time/length/volume units so we have added these throughout the manuscript. We will defer to the journal for additional spacing requirements.

*3. Ln 97: What temperature were samples stored at?*

We have added text to the Methods Section stating that the samples were stored in the refrigerator at 4°C until they were processed.

*4. Ln 186: What was the total number of fungi sent for identification and what was the number breakdown by tissue sampled (i.e., beetle wash, phloem chips)*

Text has been added to the Methods Section to indicate that a total of 54 adult beetles and 54 phloem samples were collected from across the seven sites, with sample size breakdown information provided in Fig 2 and its caption.

*5. Ln 433 (Fig. 4): Consider referring to all the panels in this figure in text – even if it is just in the Table 1.*

Fig 4A and 4E are referenced in the main text of manuscript as they relate to the physical description of the cultures therein. Since we do not describe the morphology of cultures of the other species, we have opted not to add more in-text references to the Fig 4 panels but believe that it is still appropriate to provide these panels in the manuscript (e.g., as reference information for future studies).

*PDF-embedded comments:*

*6. For a better readability I would suggest to use the form “*Neonectria *sp. nov.” instead of “*N*. sp. nov.”*

We agree and have updated *N*. sp. nov. to *Neonectria* sp. nov., as well as *O*. sp. nov. to *Ophiostoma* sp. nov., throughout the manuscript.

7*. Ln 359: Indeed not really, it was shown that hydrophobins present on the conidia surface can mediate the attachment to hydrophobic surfaces, as the chitinous exoskeleton of the insect vectors and the conidia themselves (Wösten 2001; Temple and Horgen 2000). This is the case for entomopathogenic fungi, such as* Beauveria bassiana *(Zhang et al. 2011), and* Geosmithia *(Bettini et al., 2012) where the adhesion of conidia to the host’s surface is mediated by nonspecific hydrophobic and electrostatic interactions involving hydrophobin rodlet layers on the conidial cell wall.*

We thank Reviewer #2 for these insights which are extremely relevant to our interpretation of the relationship between the alder bark beetle and the *Neonectria* fungus. We have removed “Surprisingly” from L407 as suggested but note that our emphasis was directed to the identification of a non-ophiostomatoid associate, although we acknowledge that this statement did not account for the widespread associations between *Geosmithia* spp. and bark beetles which has since been addressed elsewhere in the manuscript (see responses to Reviewer #1 points 6 and 8). We have added one of the recommended papers (Bettini et al. 2012) to our statement about *Geosmithia* and *Neonectria* fungi lacking the sticky spores characteristic of ophiostomatoids and allude to potential alternate mechanisms for spore adhesion (L423-427). We will certainly address the potential role of hydrophobins in exoskeletal spore adhesion in a forthcoming paper that focusses on alder bark beetle vectoring of *Neonectria* sp. nov. and other insect vectors of Hypocreales fungi.

**Author-initiated revisions**

Minor grammatical, clarification, and citation edits with track-changes were made to the manuscript.

Tables 1 and S1 were formatted slightly to accommodate accession numbers.
